# Modeling transcriptomic age using knowledge-primed artificial neural networks

**DOI:** 10.1038/s41514-021-00068-5

**Published:** 2021-06-01

**Authors:** Nicholas Holzscheck, Cassandra Falckenhayn, Jörn Söhle, Boris Kristof, Ralf Siegner, André Werner, Janka Schössow, Clemens Jürgens, Henry Völzke, Horst Wenck, Marc Winnefeld, Elke Grönniger, Lars Kaderali

**Affiliations:** 1 Front End Innovation, Beiersdorf AG, Hamburg, Germany; 2 Institute for Bioinformatics, University Medicine Greifswald, Greifswald, Germany; 3 Institute for Community Medicine, University Medicine Greifswald, Greifswald, Germany

**Keywords:** Molecular biology, Biological techniques

## Abstract

The development of ‘age clocks’, machine learning models predicting age from biological data, has been a major milestone in the search for reliable markers of biological age and has since become an invaluable tool in aging research. However, beyond their unquestionable utility, current clocks offer little insight into the molecular biological processes driving aging, and their inner workings often remain non-transparent. Here we propose a new type of age clock, one that couples predictivity with interpretability of the underlying biology, achieved through the incorporation of prior knowledge into the model design. The clock, an artificial neural network constructed according to well-described biological pathways, allows the prediction of age from gene expression data of skin tissue with high accuracy, while at the same time capturing and revealing aging states of the pathways driving the prediction. The model recapitulates known associations of aging gene knockdowns in simulation experiments and demonstrates its utility in deciphering the main pathways by which accelerated aging conditions such as Hutchinson–Gilford progeria syndrome, as well as pro-longevity interventions like caloric restriction, exert their effects.

## Introduction

In recent years the increasing availability of large-scale molecular biological data from high-throughput experiments, in parallel with technological advancements in machine learning and bioinformatics, have greatly accelerated the discovery of biomarkers and fueled the use of computational modeling to unravel complex biological phenomena. In aging research particularly, the discovery of the ‘epigenetic clock’—a machine learning model predicting individual age using genome-wide DNA methylation data—as a highly accurate and reliable biomarker of biological age, has understandably sparked immense interest in the research community. Since then, numerous age clocks have been developed and the concept expanded to further levels of biological data, using transcriptomic, proteomic, and metabolic features^1–9^. While no other data type thus far allowed prediction accuracies quite on par with those achievable using DNA methylation data, features based on metabolite production or gene expression are arguably causally a step closer to the aging phenotype, thereby—at least conceptually—increasing the interpretability of the biomarker. Previously published age clocks based on these data types have not been capitalizing on this conceptual advantage however. On the contrary, interpretability has frequently been neglected as a property in these models so far, no matter the type of data used.

We argue that increasing the interpretability of age clocks may unlock unprecedented utility of these machine learning models in aging research and help expand their use in applied research, e.g. in a human cell-culture-based screening setting, where finding suitable holistic cellular read-outs for the biological aging state is not an easy task and added interpretability could offer additional insight on potential mechanisms of action for given treatment approaches. The concept we propose to achieve this is based on a knowledge-primed artificial neural network, in which information on biological pathways in the form of gene-pathway annotations is incorporated into the architecture of the model. A similar approach has recently been shown to be effective in the modeling of yeast growth from transcriptomic data^10^. Normally, artificial neural networks feature densely connected layers of neural units, in which every neuron in a given layer is connected to every neuron of the next layer. As the information flow through the network is not linked to any particular processes and connections between neurons are essentially interchangeable, it is inherently hard to interpret, which is why deep learning models are frequently quoted as examples of ‘black box’ models. A defining feature of artificial neural networks however, is the flexibility they offer to implement architectures with unique properties. Omitting the fully connected design and restricting the connections between neurons as implemented for the proposed new age clock can be used to guide the flow of information within the network, thereby augmenting and controlling the way the model learns. Importantly, this allows for the embedding of prior information on biological processes, such as the pathway annotation of genes, directly into the model architecture and therefore ties the model’s learning process to known biological processes. Such a design thus enables the model to learn pathway-based representations of the molecular data, which—through the inspection of neuron activations in the pathway layers—allows the monitoring of pathway aging-states and delivers interpretability to the clock’s inner workings.

In order to evaluate the utility of this approach for aging research, we constructed a pathway-based artificial neural network and trained it for age prediction based on a large transcriptomic dataset from epidermal skin samples (*n* = 887). Skin represents an extraordinarily well-suited tissue for studying aging, owing to its well-documented aging phenotype and the ease of sampling using non-invasive procedures. As it represents the body’s outermost layer, shielding other tissues from hazardous external influence, it also offers the unique possibility to study extrinsically accelerated aging, phenotypically well-documented in the form of photoaging^11^. The data used to construct the model was derived from the latest iteration of the ongoing Study of Health in Pomerania (SHIP), SHIP-TREND, a longitudinal cohort study generating a broad population-based picture of health and disease in northeastern Germany^12^. Owing to its unbiased observational design, the study is particularly well-suited to investigate the natural aging progression.

## Results and discussion

### Architecture of the neural age clock

**Fig. 1:**
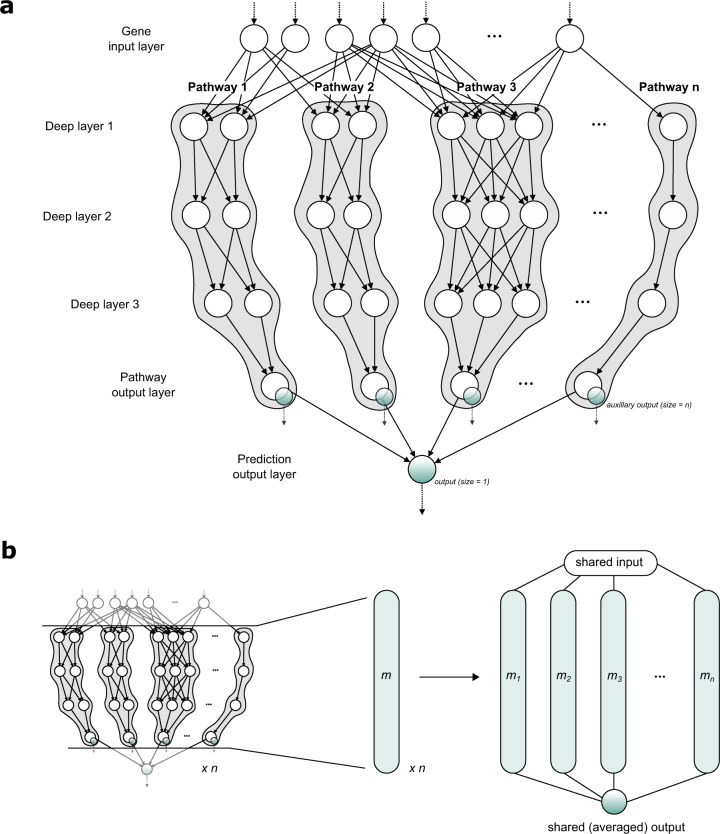
Model architecture and setup. **a** Schematic of the artificial neural network architecture. Gene expression data is fed to the input layer, which is connected to the following hidden layer through gene-specific edges that are constructed based on pathway affiliation. In the following hidden layers, information is processed by the network in a pathway-centric manner culminating into a final linear pathway layer with one neuron per pathway, which also serves as an auxiliary output to monitor pathway aging states. Finally, the information from all pathway neurons is aggregated in the main output neuron, which generates the age prediction. **b** Ensemble setup. To improve the stability and accuracy of the final model, an ensemble model was constructed from individually trained networks by joining the separate models to the common input and output layers.

The architecture of the artificial neural network was modeled based on the ‘Hallmark’ pathway collection, a selection of 50 conserved and highly refined gene sets, capturing essential biological processes, created to improve pathway inference by reducing variance and gene overlap, as it is often found in larger pathway collections such as GO terms^13^. The pathway-guided design generates a compartmentalized neural network, in which different parts of the network model distinct pathways, enabling the activations of intermediate neurons to be interpreted to generate insight on the aging states of diverse biological processes. As such the network consists of a single input layer for the gene expression data, followed by four hidden pathway layers and two separate output layers (Fig. 1a), the main output generating the final age estimate, the auxiliary output providing summarized information on the aging states of the respective biological pathways.

To improve both reproducibility and accuracy of the age clock, an ensemble learning approach was implemented. For the final model, a stacked ensemble was constructed from 10 individually trained networks, which shared input and output layers (Fig. 1b). Ensemble stacking is a popular approach to improve the generalization ability of machine learning models by combining the strengths of different model instances, such as those awarded by different weight configurations learned in individual training reboots of neural networks^14^. We found that stacking several models improved prediction accuracy by around 0.3 years, and importantly further cemented the reproducibility of the learning process.

### Model training and testing

**Fig. 2:**
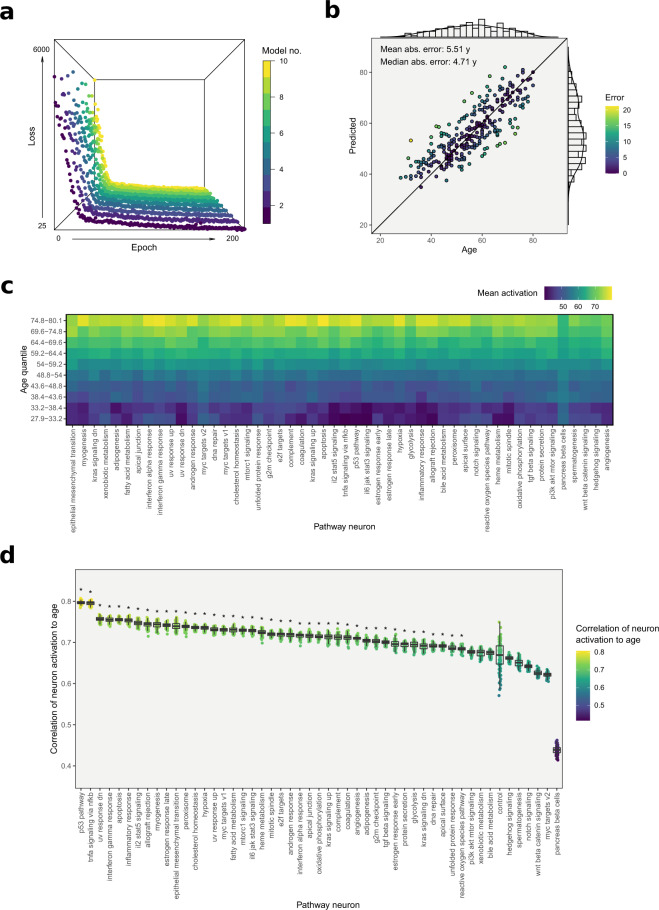
Training and performance testing of the neural age clock. **a** Training history of the 10 individual neural networks. Depicted is the loss on the held-out testing set, over the full range of 200 training epochs. **b** Predicted against actual chronological age for the held-out test set, with observations colored by absolute prediction error. **c** Heatmap showing distinct activations of pathway neurons for the test set samples stratified by age quantiles. **d** Pathway ranking based on Pearson correlation coefficients of pathway neuron activations and chronological age over the test set. The results shown are based on 100 permutations calculated for a model including an artificial control pathway consisting of 150 randomly sampled, unrelated genes as a baseline. Significance was determined using one-sided Wilcoxon rank-sum tests comparing the correlation estimates of the various pathways to the introduced control pathway, adjusted for multiple testing.

As a basis for model training, gene expression data were generated via RNA sequencing from epidermal skin samples collected from 887 subjects aged between 30 and 89 years in the SHIP-TREND cohort study (Supplementary Fig. 1a and b). The data were randomly split into independent training and test sets (70/30), with the test set of 267 samples reserved for accuracy assessment and further in silico experiments, leaving 640 samples for model training. The 10 neural networks making up the final model were trained separately for 200 epochs each (Fig. 2a) until no further substantial improvements were detectable without risking overfitting, and then combined into an ensemble by fusing their input and output layers. Assessment of the final age clock’s accuracy on the independent test set revealed a median absolute error of 4.7 years (Fig. 2b). This is similar in performance to published ‘black box’ clocks on transcriptomic data^5,7,8,15,16^, which generally tend to perform worse in terms of pure accuracy compared to their DNA methylation-based counterparts^17^. We additionally trained a fully connected “black box” neural network with a comparable number of parameters in the same ensemble approach on the same data, which slightly outperformed its pathway-based counterpart with a median absolute error of 4.4 years (Supplementary Fig. 2a). Based on our data, this suggests that there is a small trade-off between transparency and precision, albeit at a rate that might well be tolerable in practice.

### Transcriptomic age is associated with visual age estimates

As the skin presents a well-suited tissue to observe the phenotypic manifestations of aging, we investigated if the transcriptomic age estimates generated by our pathway-based age clock were associated with any phenotypic markers of age. For this, we used standardized portrait images of a random subset of 154 subjects from the test set and generated visual age estimates using a blinded expert panel, tasked to assess the age of the test subjects from the portrait photographs. Linear modeling identified a significant association between the average visual age estimates of this panel and the transcriptomic age predictions (*p* = 0.016) after adjusting for chronological age and gender (Supplementary Table 1), delivering not only a validation of the clock’s capabilities to detect biological aging state but also evidence of a direct link between phenotypic manifestations of aging and the molecular alterations in aging skin, captured by the model.

### Model reveals the wide-spread impact of aging on the global pathway landscape

Visualizing the intermediate pathway neuron activation for samples of different ages in the pathway-based age clock shows increasing activations for older subjects, allowing not only a general glimpse into the inner workings of the clock but also the detailed assessment of aging states of single biological pathways (Fig. 2c). Ranking the pathways based on a correlation analysis of the intermediate neuron outputs with the actual ages of the subjects revealed p53- and TNFa/NFkB-signaling as the pathways that most clearly captured the aging state out of all modeled processes (Fig. 2d and Supplementary Table 2). However, the margin to the rest of the pathways was rather small and most of the processes showed a significantly higher age association than an artificially introduced control pathway consisting of randomly sampled genes, indicating that the impact of age on gene expression is indeed a global phenomenon, rather than being restricted to a few pathways. The most notable exception to this finding was the low correlation of the pancreas beta-cell pathway at the other end of the spectrum. This might be explained by the low overlap in gene function between pancreas and skin however, given that this gene set mainly describes the differentiation process of beta cells.

The wide-spread impact of increasing age on biological processes meanwhile is in line with the general aging hypothesis of the deleteriome^18^. The deleteriome hypothesis attempts to unify a variety of previous theories of aging under a common motif, the eponymous accumulation of deleterious effects over the lifetime, which are amplified by the inherent imperfection of biochemical processes and reactions. The theoretical framework encompasses previously proposed theories such as the free radical theory of aging^19^ but further expands the scope to include observations and theories from evolutionary biology such as the existence of antagonistically pleiotropic genes^20^. The key feature of the theory, despite managing to unify the various explanatory approaches to how the process of aging arises, is that it importantly predicts no single ‘master switch’ gene or biological process that drives the natural aging progression, but rather a plethora of small individually detrimental alterations to cellular and organismal function accumulating over time. The model’s estimates on biological pathway relevance would seem to support this.

### In silico gene knockdowns recapitulate associations from the literature

**Fig. 3:**
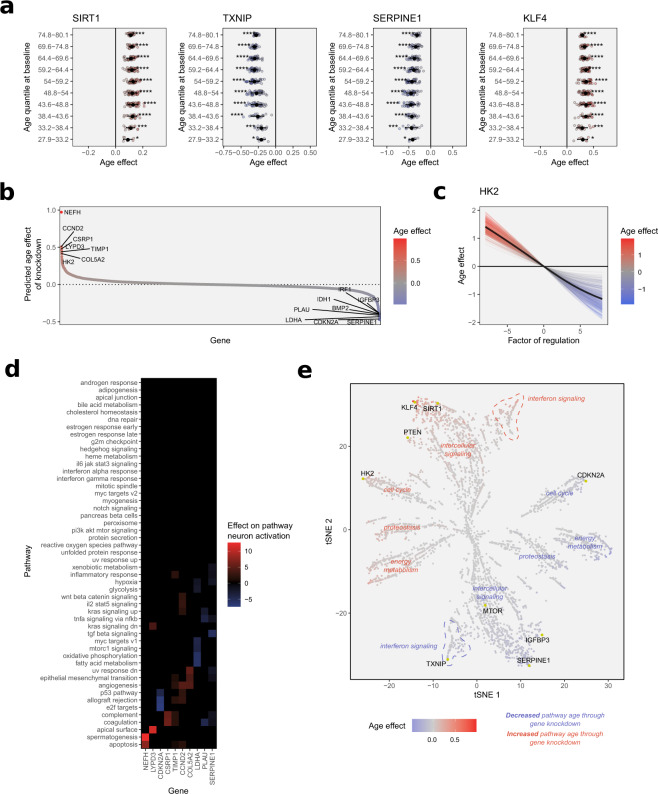
Identifying relevant aging target genes through simulated gene perturbation. **a** Predicted effects of the in silico knockdowns of SIRT1, TXNIP, SERPINE1, and KLF4. Effect on age is stratified by chronological age quantiles of the test subjects. Significance was determined using one-sample Wilcoxon rank-sum tests, testing for the difference in medians from an effect size of 0, with *p*-values adjusted for multiple testing. Error bars show standard deviations. **b** Distribution of the predicted age effects of the in silico knockdown of all genes covered by the model. Genes at the upper and lower extremes can be regarded as the most important features of the model. **c** Predicted age effect of the simulated continuous knockdown and overexpression of HK2, one of the most important genes in the model. **d** Effects of simulated gene knockdowns by the most important genes according to impact on age estimation upon the activation of pathway neurons. **e** Two-dimensional embedding of the aging pathway landscape. The map was generated by assessing the effects of all gene knockdowns on pathway neuron activation and calculating a lower-dimensional embedding of the data using the tSNE algorithm. Genes are colored by the overall impact of their knockdown on the final age estimate. Thereby clustering of genes according to strength and direction of correlation to age, as well as functional pathway annotation, can be observed.

Seeing that the performance of our clock compared reasonably well to ‘black box’ models and achieved transparency on the biological processes affected, we next set out to test how well the clock actually captured known aging mechanisms and associations through a series of in silico experiments. As discussed above, past research has not identified a single ‘master switch’ gene or pathway driving aging, nonetheless, several genes have been identified over the years, whose deregulation is associated with changes in lifespan in model organisms or the manifestation of aging phenotypes. To test if the model could recapitulate such associations, we performed virtual gene knockdowns of known aging target genes with a history of experimental data available from model organisms and human genome-wide association studies, to evaluate if the predictions accurately replicated the effects of the perturbation (Fig. 3a). The knockdown of SIRT1 for example, a widely studied NAD-dependent deacetylase with various conserved pro-longevity functions, has been shown to have detrimental effects on the lifespan of several models organisms^21–24^. Indeed, simulation of a decreased SIRT1 regulation by a log2 fold-change of −2 using our model predicted a significant age increase for all subjects in our test set, in concordance with expectations and data from the literature. In contrast, the knockdown of thioredoxin-interacting protein TXNIP, a major player in maintaining cellular redox-status and recently implied in the induction of senescence by its role in antagonizing AKT-signaling^25^, reduced predicted ages significantly, in line with experimental data that shows that knockdowns of TXNIP increase life-span by reducing reactive oxygen species (ROS)-mediated stress in model organisms^26^. Moving away from model organisms, a null-mutation of SERPINE1 is one of the few causal associations discovered so far, that links a single gene loss-of-function mutation directly with increased longevity in humans^27^. In line with the literature, simulated knockdown of the senescence-associated gene lead to a significant decrease in transcriptomic age predicted by the model. These simulations, while intended mainly as validation of the associations learned by the model, also highlight the utility of computational models for translational research, in this case, the ability to test the relevance of target genes identified in a systemic context or in other tissues to the biology of aging skin, which the model was trained on. An example of a gene association more specific to the skin however, is the knockdown of Krueppel-like Factor 4. KLF4 is, among others, a stemness factor and direct regulator of telomerase expression^28^, as well as importantly a regulator of keratinocyte senescence^29^. As such, KLF4 silencing alone has been shown to be sufficient to induce a senescent phenotype in human keratinocytes^29^. In line with these findings, the simulated knockdown resulted in an increased age prediction across subjects of all ages.

### Systematic knockdown simulations identify known and novel aging target genes

As the knockdowns of selected literature-based aging target genes had recapitulated experimental findings, we then extended the knockdown to the rest of the transcriptome, at least insofar as it was covered by the Hallmarks pathway annotation database and therefore represented in the model. Simulating the knockdown of all genes by a log2 fold-change of −2 revealed an approximately equal distribution of age increasing and decreasing knockdowns, ranging from around +1 to −0.5 years in effect sizes (Fig. 3b). Among the highest-scoring knockdowns of all genes were several well-described aging marker genes, such as SERPINE1, IGFBP3, CDKN2A, and TIMP1, as well as some less intensely studied genes such as HK2, a hexokinase whose expression has previously been reported to diminish with increasing age in the skin, with potentially detrimental effects on energy metabolism and epidermal cell proliferation^30^. The simulated overexpression of HK2 on the other hand was concordantly predicted by the model as a rejuvenating intervention (Fig. 3c), highlighting the utility of interpretable machine learning models to discover novel angles and targets for potential intervention strategies.

Observing the effects of the most influential gene knockdowns on pathway neuron activation revealed that interestingly all of them mediated their effect via at least two distinct pathways (Fig. 3d), indicating that genes at the crossroads of several pathways might exert a larger influence on the final age estimate, which was confirmed by association testing (Supplementary Fig. 3a) for both positive (*p* = 2.6e−116) and negative impact genes (*p* = 2.4e−153). This indicates that the network architecture organically increases the impact of master regulators and genes which act as effectors in several different biological processes. This emergent property is very much desirable, as it reflects the underlying biology more closely than other machine learning models that tend to weight features purely based on predictivity or correlation to the modeled phenotype, rather than by the breadth of their biological impact. We subsequently expanded the pathway impact analysis to all genes covered by the model and found that using the single-gene knockdown data allowed reconstruction of the aging pathway landscape, with genes arranged by similarity in effect as well as capturing the structure of the diverse biological motifs and processes. The resulting map (Fig. 3e) demonstrates the gain in interpretability awarded by this new type of clock, allowing the visual inspection of gene–pathway relationships in the context of aging, unlike any previous age clock.

### Predicting the impact of complex transcriptional signatures on biological aging state

**Fig. 4:**
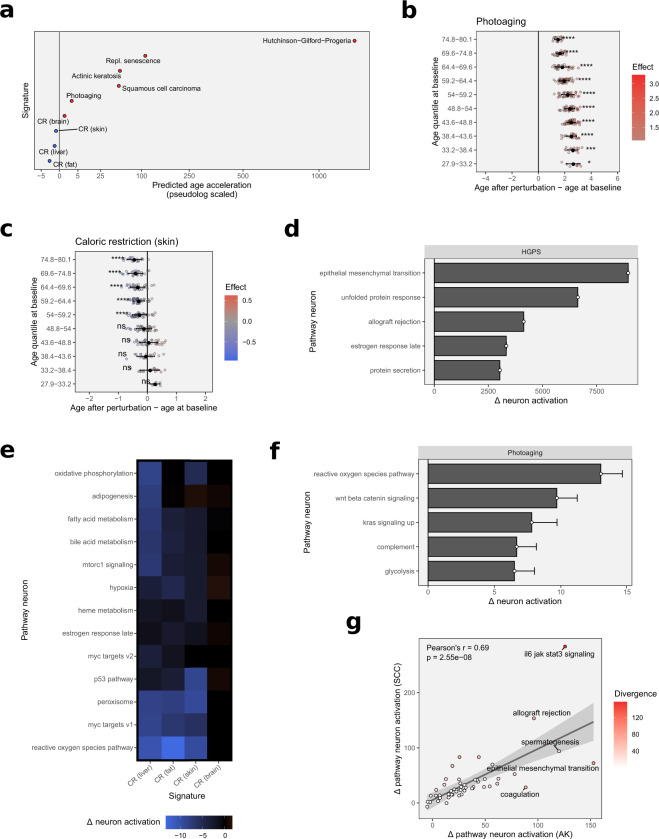
Assessing the impact of age- and disease-related gene expression signatures on transcriptomic age and pathway aging states. **a** Overview of the average predicted effect of the transcriptomic perturbation using multiple age- and disease-related signatures. **b** Predicted effect of the transcriptomic perturbation using a signature of chronically sun-exposed skin, stratified by chronological age quantiles. Significance was determined using one-sample Wilcoxon rank-sum tests, testing for the difference in medians from an effect size of 0, with *p*-values adjusted for multiple testing. Error bars show standard deviations. **c** Predicted effect of the transcriptomic perturbation using a caloric restriction signature, stratified by chronological age quantiles. Significance was determined using one-sample Wilcoxon rank-sum tests, testing for the difference in medians from an effect size of 0, with *p*-values adjusted for multiple testing. Error bars show standard deviations. **d** Effect of the transcriptional signature of Hutchinson–Gilford progeria syndrome on pathway neuron activation. Shown are the five most strongly affected pathways. Error bars show standard deviations. **e** Heatmap showing the effects of tissue-specific caloric restriction signatures on pathway neuron activation. **f** Effect of the transcriptional signature of photoaging on pathway neuron activation. Shown are the five most strongly affected pathways. Error bars show standard deviations. **g** Impact of actinic keratosis (AK) and cutaneous squamous cell carcinoma (SCC) signatures on pathway neuron activations.

We then set out to evaluate the impact of more complex aging-related transcriptional signatures on model prediction. This analysis served two purposes: (i) investigate if the model recapitulates the overall effect of the signature and (ii) demonstrate the use of an interpretable machine learning model in deciphering the biological processes driving accelerated aging or rejuvenating conditions. For this, we searched the literature for gene expression data or published signatures of diverse aging-related conditions and simulated their impact on the predicted age of the test set (Fig. 4a).

The most prominent example of an accelerated aging disorder is the Hutchinson–Gilford progeria syndrome (HGPS). HGPS is a rare autosomal dominant genetic disorder that manifests very early in life, with symptoms that strikingly resemble those of natural aging particularly in regards to the skin, including wrinkle formation, the emergence of dyspigmentations (age spots), and a general thinning of the skin including a loss of subcutaneous fat, as well as alopecia^31^. The condition is caused by mutations leading to incorrectly processed forms of lamin A that weaken the nucleus structure with diverse detrimental consequences. The overall effects of this are severe, and the average life expectancy for patients is only between 13 and 15 years^31,32^. Simulating the effect of the transcriptomic signature of HGPS^33^ likewise has a heavy impact on age estimation, with the clock putting out predictions beyond 1200 years after signature application (Fig. 4a). Though these numbers might at first seem absurdly high, they are easily explained considering the clock was trained on data of a natural aging progression. The fact that predictions are exceeding this scale is caused by the underlying learned mathematical model and signals that, while the model clearly assesses HGPS or aspects of HGPS as an accelerated aging condition, the transcriptomic state seen in HGPS is shifted far beyond that of the natural physiological aging progression. The effect size can therefore be interpreted as a manifestation of the pathophysiology of the underlying condition, in sync with the low life expectancy of individuals suffering from HGPS.

A milder form of accelerated aging, one that specifically affects the skin, can be observed in the form of photoaging. Caused by the chronic exposure of the skin to solar irradiation, photoaging is an extrinsically accelerated aging phenotype, characterized by wrinkling, dyspigmentation, and a leathery appearance of the skin^11^. Simulating the impact of the signature of chronically sun-exposed skin^34^ increases the predicted age by around 2.1 years on average (Fig. 4b). This result is again in line with expectations but importantly demonstrates that the clock is sensitive enough to be used to detect smaller transcriptional alterations caused by exogenous stressors that affect aging, such as chronic sun exposure.

Further unprotected from the damages of solar irradiation, photoaged skin can over time develop into scaly pre-cancerous lesions known as actinic keratoses (AKs). AKs, caused by the intraepidermal proliferation of atypical keratinocytes, are a frequently diagnosed skin condition in light-skinned individuals with a history of sun exposure^35^. Although themselves often asymptomatic, around 10% of all AK lesions progress into cutaneous squamous cell carcinoma (SCCs) if left untreated^35–37^, one of the most common types of cancer in developed countries with predominantly fair-skinned populations^38^. Due to the direct link between photoaging and the emergence of AKs, and the direct progression path from AKs to SCCs, we decided to include signatures from these pre-cancerous and cancerous tissues into the analysis. Interestingly, both signatures^39^ induced substantial increases in predicted age across all samples, on average by 54 and 52 years, respectively (Fig. 4a). Considering the hyperproliferative traits of both disorders this might appear somewhat counter-intuitive, then again, the relationship between aging and cancer is complex, and several shared mechanisms between the two have been identified over the years^40^, let alone the fact, that age remains one of the greatest single risk factors for the development of cancer overall^41^.

A key feature of aging that is lately receiving increasing attention, and also happens to play an important role in tumorigenesis, is the accumulation of senescent cells in aging tissues. Likely evolved as a cancer protection mechanism, senescence describes the cessation of cell division, induced by extrinsic stress or replicative exhaustion. Senescent cells influence their surrounding tissue by secreting a complex proinflammatory mixture of cytokines, growth factors, and proteases^42^. This senescence-associated secretory phenotype (SASP) plays an important role in the recruiting of immune cells to the tissue, and as such has beneficial functions in wound healing and tissue regeneration^43^. In aging tissues however, the increasing accumulation of senescent cells impairs normal tissue function, and SASP has been proposed as one of the mechanisms that drive inflammation, the chronic low-grade inflammatory state of aging tissue^44,45^. As senescence is an important aspect of aging and also a common in vitro model of aging, we tested the signature of replicative exhaustion-induced senescence using the model. The simulations showed an increase in age of over 100 years on average (Fig. 4a), which is the strongest impact of any signature we recorded apart from HGPS. It should be noted here, that previous experiments calculating the DNA methylation age of fibroblasts in culture have estimated cells aging around 62× faster in vitro^46^, which could factor into these predictions as well. Irrespective of this, the data shows that the clock not only accurately captures aging in vivo but also models processes that define aging in vitro, adding to its utility. The sensitivity of the model towards senescence also delivers one potential mechanism explaining the pronounced age acceleration predicted by the model for the AK and SCC signatures, as an accumulation of senescent cells is not only a feature of aging tissues but also frequently observed in precancerous and cancerous lesions, including AKs and SCCs^47,48^.

Next, we were interested in seeing if the model was also capable of recapitulating the positive effects of lifespan-extending intervention strategies. Most data on pro-longevity interventions stem from experiments with model organisms, but one of the advantages of the presented in silico approach is the opportunity to transfer such settings into a human model and simulate the effects of such treatment in human tissue. The most reliable and well-documented form of pro-longevity intervention is caloric restriction^49^. The reduction of caloric intake has been shown to increase health- and life span in a large number of organisms of varying size and complexity, including roundworms, flies, mice, rats, and even non-human primates^50^. It is therefore believed to be a conserved mechanism among animals, although its effectiveness in terms of lifespan extension has yet to be proven in humans. Data from model animals are generally amply available, we did however only identify a single recently published dataset that included the transcriptional patterns triggered by caloric restriction in skin tissue, which was based on *Rattus norvegicus* samples^51^. Mapping the gene signatures from this dataset to their human homologs allowed testing the signature with the age clock and simulate its effects on human aging. The signature indeed shifted the aging transcriptome landscape to a younger state by around 0.2 years on average, although the effect was only statistically significant for subjects above 50 years (Fig. 4c). Despite its low effect size, this indicates that caloric restriction might indeed have beneficial effects in humans, and ones that might favorably affect skin biology. The data also points to the existence of an age-dependency of these effects, a theory that has interestingly been proposed before and is backed by experimental data from mice showing that the beneficial impact of the intervention, while significant in adult animals, is lacking in younger specimens^52^. Conceptually this has been explained with caloric restriction largely mediating alterations to biological processes that accumulate throughout age, therefore lacking an impact on young organisms, when these processes still operate smoothly, and scarcity is more likely to impair normal functioning^53^. The age-dependency predicted by our model would further seem to support these hypotheses. As most molecular analyses of the effects of caloric restriction have been performed in other tissues though, we expanded our simulations to the signatures generated from liver, fat, and brain tissue^51^. The predicted rejuvenation of both liver, as well as fat signatures, was greater, reducing age estimates by 0.4 and 1.5 years, respectively (Fig. 4a). As these tissues are more immediately involved with and affected by caloric restriction schemes, this appears plausible. Surprisingly however, the brain signature lead to divergent results and caused the model to predict a small but significant age acceleration by 0.4 years on average. While this may simply be an artifact of tissue-specific gene regulation, one might speculate on the involvement of a biological component as well. Being the most demanding organ in terms of energy needs in most animals, it is conceivable that the brain would be the organ most immediately affected by negative repercussions of decreased caloric intake, which could help explain the finding. This theory is supported by data from non-human primates under caloric restriction, that—despite showing significant life-span extension—suffered from an accelerated loss of gray brain matter, albeit without affecting cognitive performance^54^.

### Decoding the pathways implicated in accelerated aging and pro-longevity phenotypes

Seeing that the model was capable of recapitulating both accelerated aging and pro-longevity interventions in the form of caloric restriction, we were interested in establishing the network’s utility in deciphering the biological processes by which these conditions exerted their effects. For this, we analyzed the activations of the pathway neurons in the intermediate pathway output layer before and after perturbation with the respective signatures and monitored the changes induced in neuron activation.

The most substantial alterations to the pathway landscape were caused by the transcriptional signature of HGPS (Fig. 4d). The effects were dominated by a massively increased positive activation in the epithelial–mesenchymal transition pathway neuron, indicating a substantial shift in pathway states towards an older transcriptome, but far surpassing the originally modeled range. Epithelial–mesenchymal transition describes the process of epithelial cells losing their polarity and gaining functions allowing them to migrate and gain mesodermal character. This process, while originally observed during embryogenesis, has since been shown to be a crucial mechanism in the metastasis of cancers, during wound healing, and—importantly—in the manifestation of fibrosis^55,56^. The cause of death in patients suffering from HGPS is usually found in cardiovascular complications from substantial levels of atherosclerosis, but interestingly in the absence of typical risk factors such as increased L-LDL or C-reactive protein^57^, and with more prominent signs of vascular fibrosis than typically observed in patients suffering from cardiovascular disease^58^. Interestingly then, the most strongly affected pathway identified by the model is one with a direct connection to the most severe clinical feature of HGPS, which might warrant further investigation, especially since this pathway has not received a lot of attention in studying the disease progression of HGPS thus far. Other noteworthy pathways that were strongly affected by the signature were related to proteostasis and protein secretion, immune signaling, and the estrogen response (Fig. 4d), several of which are not only well described Hallmarks of Aging^59^ but have also previously been associated with HGPS^60^.

In contrast to the HGPS signature, analyzing the pathways impacted by caloric restriction revealed a number of processes shifted towards a younger state (Fig. 4e). The effects were generally similar between tissues, with the exception of the brain-derived signature, which showed no substantially rejuvenated pathways at all. The processes that were most prominently shifted towards a favorable state were related to ROS, peroxisome pathways, and to a lower extent mTOR-signaling and general metabolism across all tissues. Reduced production of ROS through a slowing of the metabolic rate, thereby reducing the load of oxidative stress, is one of the very key mechanisms proposed by which caloric restriction is believed to exert its life-span extending effects, the observed changes in pathway states are therefore very much in line with existing theories and reports^61,62^. Another well-described effect of restricting caloric intake is the reduction of mTOR activity, marking one of the most reliable single mechanisms to prolong lifespan in various model organisms from fruit flies to non-human primates^63–65^. The rejuvenating impact on mTOR-signaling predicted by the model is therefore again very much in line with existing data, as are naturally the observed effects on metabolic pathways, including oxidative phosphorylation and fatty acid oxidation in mitochondria and peroxisomes. Interestingly though, the skin-derived signature appeared to have a lower impact on metabolic pathways but instead showed a more strongly rejuvenated profile associated with p53-signaling, which is an interesting finding considering its crucial role in cancer protection in the skin^66^. Notably, caloric restriction has been shown to delay carcinogenesis and tumor-related mortality in rodents^67,68^ and rhesus monkeys^69,70^, this finding could therefore be suggestive of another potential benefit of caloric restriction for skin biology. It should be noted that as these results represent a translation from rodents to human biology, so a margin of error is to be expected. The analysis does however highlight the potential of interpretable machine learning to use available data from animal experiments and to explore the translation of findings to a model of human biology in a virtual setting.

The effects of the photoaging signature were similarly diverse, with the strongest impact also recorded on the ROS pathway (Fig. 4f), here substantially shifting the pathway towards an older state. Further processes altered in this direction were related to Wnt and Kras signaling, and metabolic pathways such as glycolysis. Interestingly a couple of pathway states appeared shifted towards a younger profile, most notably involving the G2 damage checkpoint and the estrogen response pathways. The effects of a chronic exposure to solar irradiation that over time lead to the manifestation of photoaging, are believed to be primarily driven by oxidative damage resulting from the UV-induced formation of ROS^11,71–73^. The predominant pathway identified by the model very much supports this hypothesis. Metabolic changes in photoaged skin have likewise been reported^34^. Data on Wnt modulation in association with photoaging is sparser, but recent reports implicate the pathway in the response following UVB irradiation in keratinocytes in vitro^74^. Given its function as an important mediator of cell proliferation and differentiation and importantly its essential role in regulating adult epidermal stem cell reservoirs, regulatory alterations in Wnt signaling could potentially be an important mechanism driving the gradual thinning of the epidermis frequently observed in (photo-)aged skin^11,75^.

Finally, we investigated the similarity in pathway neuron activation following perturbation using the AK and SCC signatures. Although the progression from AKs to SCCs, in general, is well-described, only around 10% of all AK lesions develop into actual carcinoma^35–37^. The exact mechanisms determining which AKs progress meanwhile remain elusive. Analyzing the predicted pathway perturbations revealed a substantial correlation between pathway patterns induced by AK and SCC signatures (Fig. 4g). Given the reported progression path, this finding seems conclusive. The analysis also revealed a number of pathways that were notably more strongly deregulated than others, mainly related to IL6-JAK-STAT-signaling, immune pathways and coagulation, a gene set that contains many genes related to the complement system as well as senescence-associated genes such as SERPINE1. The latter is particularly interesting, as the prolonged expression of the senescence marker gene CDKN2A has very recently been shown to induce hyperplasia in the epidermis of mice very similar to the early stages of AKs by increasing proliferation of surrounding keratinocytes, implicating senescent cells as one of the early mechanisms in epidermal tumorigenesis^76^. The comparably lower activation in the SCC signature suggests that the impact of senescence-associated genes is higher in the early stages leading to AK lesions though, which fits the experimental data available^76^. Among the processes that showed notable divergences between AKs and SCCs as well were immune and JAK-STAT-signaling, both found more strongly altered by the SCC signature. The involvement of immune-related genes contained in the allograft rejection gene set is of little surprise given that alterations to immune signaling in cancer are well-documented, the increased activation induced by the SCC signature does however highlight a very important characteristic of SCCs, which is their ability to evade immune surveillance, setting it apart from pre-cancerous AK lesions^77^. Aberrant activation of JAK-STAT-signaling is a frequently reported feature in human cancers as well^78^, and SCCs are no exception^79^. Constitutive activation of STAT3 has in fact been shown to be a key event in the SCC tumorigenesis^80^, validating the model’s predictions. Surprisingly little is known about the state of the IL6-JAK-STAT axis in AKs however and seeing the diverging pathway patterns uncovered by our model and the documented importance of the pathway in tumorigenesis would therefore encourage further investigations into this pathway in AK lesions to help explain the observed heterogeneity in AK to SCC progression.

Despite their popularity and unquestionable utility as biomarkers, age clocks have thus far generated little insight into the processes that actually drive the aging progression or provoke phenotypical manifestations of biological aging. Here we present a new type of age clock, that delivers unprecedented interpretability to its inner workings. Through the incorporation of prior information on pathways into the structure of the model, the learning process is tied to known biological processes, allowing their states to be interpreted in the activation of intermediate neurons in the neural network. While not surpassing other age clocks in terms of sheer accuracy, the model’s performance is comparable with other published as well as a ‘black box’ transcriptomic age clock trained on the same data and offers greatly expanded utility beyond the use as a readout tool. We would argue that this property is more desirable in a research setting than mere predictivity and would like to see more efforts to increase the interpretability of machine learning models applied in aging research and biological research in general. Neural networks, in particular, present themselves as a very promising technology to fully unlock the potential of such approaches in an area of research that, due to the inherent breadth and complexity of the biological processes involved and ever-increasing amounts of high-throughput data available, is predestined to benefit from further technological advancements in machine learning.

## Methods

### Study of Health in Pomerania (SHIP)

SHIP was designed as a population-based study to assess the prevalence and incidence of common clinical diseases, subclinical disorders, and risk factors among the population of the Federal State of Mecklenburg/West Pomerania in Northeastern Germany^12^. Examinations of the original cohort of 4308 randomly sampled subjects between 20 and 79 years started in 1997, with two follow-up examinations being performed after intervals of 5 and 11 years. The second cohort (SHIP-TREND), comprising another random sample of 4420 adults aged 20–79 years, started in 2008, again designed with regular follow-ups. The data used in this study consisting of 887 epidermal samples were collected during the first follow-up of the SHIP-TREND cohort, with subjects aged between 30 and 89 years (Supplementary Fig. 1a and b). The study was approved by the ethics committee of the University Medicine Greifswald (ethics approval number BB 39/08). All participants signed an informed consent form and all investigations were undertaken in accordance with the ethical principles outlined in the Declaration of Helsinki.

### Tissue sample preparation

The suction blister method applied in this study has been approved by the Ethics Commission of the University of Freiburg (general approval December 8, 2008; Beiersdorf AG No. 28857). Suction blisters of 7 mm diameter were taken from the volar forearms of all subjects as previously described^81^.

### Nucleic acid extraction

As previously described^16^, tissue samples were suspended in the respective lysis buffers for DNA or RNA extraction and homogenized using an MM 301 bead mill (Retsch). DNA was then extracted using the QIAamp DNA Investigator Kit (Qiagen) according to the manufacturer’s instructions. RNA was extracted using the RNeasy Fibrous Tissue Mini Kit (Qiagen) according to the manufacturer’s instructions.

### Transcriptome sequencing

Transcriptome libraries were prepared using the TruSeq Library Prep Kit (Illumina) and sequencing performed at 1×50 bp on Illumina’s HiSeq system to a final sequencing depth of 100 million reads per sample. Sequencing data were processed using a custom pipeline including Fastqc 0.11.7^82^ for quality control, Trimmomatic 0.36^83^ for trimming, and Salmon 0.8.1^84^ for read mapping against the GRCh38 build of the human transcriptome and read quantification in the form of transcripts per million (TPM).

### Pathway-based neural network architecture

The network was implemented using keras^85^ with a tensorflow^86^ backend and fully coded in R 3.6.1^87^. In the following and for the purpose of this work, we will use the term “pathway” to denote any gene sets or knowledge-guided collections of genes involved in distinct biological processes. In order to embed this pathway information into the network, first a binary ‘gene × pathway’ filter matrix was constructed based on gene annotations to the Hallmark pathway collection^13^. This filter matrix was used to set the crucial gene-specific connections between input neurons and the neurons in the first pathway layer. The following hidden layers operated in a pathway-centric manner. Neurons assigned to the same pathway were densely connected to each other to allow the network maximum flexibility to process and learn pathway representations from the data, while no connections to neurons of other pathways were allowed, as this would break the chain of interpretability. Information of each pathway was then aggregated in a final neuron, serving a dual purpose as both a step to condense the pathway information in one neuron per pathway and as an auxiliary output of pathway neuron activations to update the network loss during training and for further analysis purpose during inference. Finally, this pathway output layer was connected to a common output neuron in the last layer, tasked with aggregating the information passed by the pathway neurons to a final age estimate. The number of neurons within the hidden layers was adjusted to the number of genes in each pathway and thus determined for every pathway individually as shown in Eq. (1):

1 $${\rm {number}}\,{\rm {of}}\,{\rm {neurons}} = 5 + \left( {\frac{{{\rm {number}}\,{\rm {of}}\,{\rm {genes}}}}{f}} \right)$$

This established a minimal size of 5 neurons per layer for each pathway, with additional neurons awarded with increasing pathway size to accommodate an increase in regulatory complexity. The neuron scaling factor *f* that determined the number of neurons added per additional gene was set to 2 in the final model (Supplementary Fig. 5a). The number of hidden layers was set to 4, as testing with more layers showed no additional gains in accuracy justifying a further increase in complexity (Supplementary Fig. 5b). Taken together, this setup resulted in a final network with 1,740,858 trainable parameters. In order to improve generalization ability and control overfitting of the model, dropout layers were inserted between the hidden layers, randomly dropping connections between the hidden layers in the training phase. Furthermore, global weight decay (regularization factor = 0.01) was implemented as another form of regularization, improving generalization ability of the model.

The model used ‘elu’ (exponential linear units) activation functions^88^ in all hidden layers, and was accordingly initialized using the He-initialization, a weight initialization scheme optimized for ‘relu’-like activation functions^89^.

The loss function for model training combined two individual losses, calculated from the mean squared error (MSE) of the main and auxiliary outputs of the network, joined together by a balancing hyperparameter *alpha* as shown in Eq. (2):

2 $${\rm {loss}} = \left( {1 - alpha} \right) \ast {\rm {MSE}}_{{\rm {main}}} + alpha \ast {\rm {MSE}}_{{\rm {auxiliary}}}$$

The advantages of this are two-fold: (i) It forces all parts of the network to be trained, ensuring that the all encoded information is utilized, and all pathway neurons are active. This is critical, as early testing showed that without the added auxiliary loss, the network would heavily rely on only one or few pathways, the selection of which varied greatly depending on initial weight configuration (Supplementary Fig. 4a). This resulted in very poor reproducibility between network reboots and only a fraction of the available information being utilized. (ii) All pathway neurons now generate a positive continuous output, which is essentially an age estimate based on the information encoded in the pathway or ‘pathway age’. This has clear benefits for the interpretability of the neuron activations, whose scale and direction could otherwise vary greatly between network reboots and which stabilized significantly through the addition of the auxiliary loss (Supplementary Fig. 4b). *Alpha* was set to 0.4 in the final model after testing different configurations (Supplementary Fig. 5c).

The training of the model was performed using stochastic gradient descent with Adam^90^ and a learning rate of 0.001, with a mini-batch size of 16 samples for a total of 200 epochs. Table 1 summarizes the parameters of the pathway-based neural network.

**Table 1:** Pathway-based neural network parameters.

Parameter	Value
Number of input genes	4359
Number of input pathways	50
Number of parameters	1,740,858
Activation function	elu
Weight initialization	He
L2-regularization (weight decay)	0.01
Dropout rate (drop probability)	0.1
Loss calculation	Mean squared error (main and aux. output)
Hyperparameter alpha	0.4
Optimizer	Adam
Learning rate	0.001
Mini-batch size	16
Training epochs	200
Training samples	620
Test samples	267

### Ensemble setup

In order to further improve both reproducibility and accuracy of the model, the final setup was designed as an ensemble of several individual networks. For this, 10 single networks were trained separately, and then joined to a common input layer and a shared main and auxiliary output. In the shared output layers, individual outputs by the 10 networks are averaged to generate the final model estimates. The ensemble setup proved successful in further stabilizing the intermediate neuron activations and thereby improving reproducibility (Supplementary Fig. 4c).

### Fully connected neural network

To assess any potential trade-off between transparency and model precision, we trained an ensemble of 10 fully connected neural networks with the same number of layers per network, a comparable number of parameters, trained for the same number of epochs on the same data with the same training/test split as used for our pathway-based model. Table 2 summarizes the parameters of the fully connected neural network.

**Table 2:** Fully connected neural network parameters.

Parameter	Value
Number of input genes	4359
Number of neurons per hidden layer	[350,350,350,50]
Number of parameters	1,789,301
Activation function	elu
Weight initialization	He
L2-regularization (weight decay)	0.01
Dropout rate (drop probability)	0.1
Loss calculation	Mean squared error
Optimizer	Adam
Learning rate	0.001
Mini-batch size	16
Training epochs	200
Training samples	620
Test samples	267

### Assessment of visual age and association analysis

In order to generate estimates of phenotypic aging state to compare with the transcriptomic age estimates by the model, we used portrait images of 154 randomly sampled subjects from the test set. The images were captured in a standardized setup, taking evenly lit (through the use of a flash diffuser), non-polarized and color-controlled frontal portrait images of the test subjects with their eyes closed, any hair (except facial hair) covered to reduce the impact of features unrelated to the skin, and any make-up removed beforehand. The images were then presented to a blinded panel of 31 experts that were asked to estimate the ages of the subjects based on these photographs. The individual age estimates were then averaged over the panel, which resulted in the final visual age estimates, which showed generally very good concordance with chronological ages with a median absolute error of 4.38 years. Linear models were then employed in R^87^ to test for an association between transcriptomic and visual age estimates, whilst adjusting for chronological age and gender (Supplementary Table 1).

### In silico gene knockdown and overexpression experiments

The perturbation of single genes was performed by up- or downregulating gene expression by a common log2 fold-change (which was −2 for all knockdown experiments, unless otherwise specified) in all samples of the test set (*n* = 267) and comparing the model’s predictions with the unperturbed baseline predictions per sample. For the assessment of age impact, the changes in the main output neuron generating the overall age estimate were analyzed. For assessing the impact on the aging state of the biological pathways, the activity of the auxiliary output neurons was monitored instead, and the generated outputs of these neurons were similarly analyzed by comparing the ‘pathway age’ estimates with the unperturbed baseline estimates per sample.

The map of the aging pathway landscape shown in Fig. 3e was generated by embedding the perturbation effects from all gene knockdowns on each of the auxiliary pathway neurons using t-distributed stochastic neighbor embedding (tSNE) into two new dimensions^91^, using the implementation of the algorithm in the routine R package^92^.

### Mapping *Rattus norvegicus* genes to human homologs

*Rattus norvegicus* genes from the caloric restriction signatures (genome build Rnor_6.0) were mapped to their human homologs (genome build GRCh38) using the biomaRt R package^93^.

### Perturbation experiments using complex gene expression signatures

Assessing the impact of more complex transcriptional signatures was performed by up- or downregulating each significantly differentially regulated gene (cutoff was an FDR < 0.05) in the signature by the exact effect size (determined by its log2 fold-change) recorded by the differential gene expression analysis. The analysis was again performed using all samples of the test set (*n* = 267) and comparing the predictions of the perturbed data with the unperturbed baseline predictions per sample, as with the single gene knockdowns. Significance of impact was determined using one-sample Wilcoxon rank-sum tests, testing for the difference in medians from an effect size of 0. When more than one comparison was performed, *p*-values were adjusted for multiple testing using the Holm–Bonferroni method^94^. Table 3 shows a summary of the signatures used for the perturbation experiments.

**Table 3:** Signatures used for perturbation experiments.

Signature	Species	Tissue	Technology	Ref.
Photoaging	*Homo sapiens*	Skin	Microarray	^34^
Hutchinson–Gilford Progeria	*Homo sapiens*	Skin	Microarray	^33^
Replicative senescence	*Homo sapiens*	Skin	RNA seq	^99^
Actinic keratosis	*Homo sapiens*	Skin	RNA seq	^39^
Cutaneous squamous cell carcinoma	*Homo sapiens*	Skin	RNA seq	^39^
Caloric restriction	*Rattus norvegicus*	Skin	RNA seq	^51^
		Liver		
		Fat		
		Brain		

### General data analysis and visualization

Data analysis in R^87^ further included the usage of the packages data.table^95^ and dplyr^96^ for data handling, as well as the packages ggplot2^97^ and ggpubr^98^ for data visualization.

### Reporting summary

Further information on research design is available in the Nature Research Reporting Summary linked to this article.

## Supplementary information

Supplementary Information

Reporting Summary

## Data Availability

Due to German and EU privacy legislation and the sensitive nature of the SHIP data, the RNA sequencing data generated in this study are available from the SHIP consortium^12^ upon official request only (https://www.fvcm.med.uni-greifswald.de/dd_service/data_use_intro.php). For any questions or assistance with the process of accessing the data please contact the consortium via transfer@uni-greifswald.de.

## Code availability

All code used to generate the results shown in this study is available for research purposes from the corresponding authors upon request.
