# Supplementary material for: Modeling transcriptomic age using knowledge-primed artificial neural networks
Source: NPJ Aging Mech Dis. 2021 Jun 1;7:15. doi: 10.1038/s41514-021-00068-5 (PMC8169742; doi:10.1038/s41514-021-00068-5)
Supplement: Supplementary file 1 — Supplementary Information [file 41514_2021_68_MOESM1_ESM.pdf]

Modeling transcriptomic age using knowledge-primed artificial neural networks

Nicholas Holzscheck[1,2], Cassandra Falckenhayn[1], Jörn Söhle[1], Boris Kristof[1], Ralf Siegner[1], André Werner[3], Janka Schössow[3], Clemens Jürgens[3], Henry Völzke[3], Horst Wenck[1], Marc Winnefeld[1], Elke Grönniger[1] and Lars Kaderali[2].

Supplementary Information:

In order to validate the functionality and reproducibility of the neural network ensemble, several additional experiments and comparisons were performed and are detailed in the following.

Comparison with a fully connected neural network

In order to compare the transparent model's performance to a comparable "black box" model, we trained an ensemble of fully connected neural networks with the same number of layers per network, comparable number of parameters per network (1,789,301 parameters from [350,350,350,50] neurons), in the same 10 network ensemble-setup, trained on the same data and training/test split as used in our pathway-based model. The accuracy reached by this "black box" model on the independent test fraction slightly outperformed the pathway-based model with a median absolute error of 4.4 years (Supplementary Figure 2 a), versus 4.71 years for the pathway-based model, with mean absolute errors even closer. This indicates that the loss in precision introduced by the pathway-based modeling approach is relatively small. Given the interchangeability of neurons connections in the fully connected network, interpretability of neuron activations is naturally no longer given in this model and intermediate layer neuron activations are no longer reproducible either, as is revealed by a comparison of activation patterns from the last 50-neuron-layer from individual networks of the ensemble (Supplementary Figure 2 b). We also repeated the knockdowns of known aging target genes with the "black box" model. The results

[1] Front End Innovation, Beiersdorf AG, Hamburg, Germany.
[2] Institute for Bioinformatics, University Medicine Greifswald, Greifswald, Germany.
[3] Institute for Community Medicine, University Medicine Greifswald, Greifswald, Germany.

match quite well with those generated by the pathway-based model (Supplementary Figure 2 c) and only differ slightly in the magnitude of impact predicted. This is interesting, as it suggests that irrespective of the network design, the models appear to learn a similar correlative structure from the data, with the notable difference of course being the interpretability of the neuronal activation patterns, which is only given using the pathway-based architecture.

Comparison of transcriptomic age and visual age

The aging phenotype of the skin is a sum of many factors, prominently including the formation of coarse and fine wrinkles, a loss of structural rigidity and elasticity, as well as changes in texture and pigmentation. The human eye is trained well on the detection of these markers, so we resorted to the use of portrait images in order to bridge the gap between molecular parameters and phenotypic manifestations of aging and validate the predictions of the model. The images were captured in a standardized setup, taking evenly lit (flash diffuser), non-polarized and color-controlled frontal portrait images of the test subjects with eyes closed, any hair covered to reduce the impact of features unrelated to the skin (except facial hair for men), and naturally any make-up removed beforehand. To enable a comparison with the transcriptomic age predictions, we generated estimates of visual age for a subset of 154 subjects from the test set from these images, by asking a group of 31 volunteers to assess the ages of the test subjects from the photographs. Averaging the predictions from all volunteers revealed a strong correlation of the estimated visual ages to the chronological ages of the test subjects, with a median absolute error of only 4.38 years. Subsequent analysis using linear models (Supplementary Table 1) identified a significant positive association between the predicted transcriptomic age and the visual age assessments after adjusting for chronological age and gender of the test subjects ($p = 0.016$). This delivers evidence of a direct link between the molecular aging state of the skin as captured by the model and tangible phenotypical manifestations of skin aging.

Supplementary Table 1: Statistics from a linear model testing for an association between transcriptomic age estimated by the neural network and visual age, assessed from portrait images, adjusted for chronological age and gender:

	Dependent variable:
	Transcriptomic age
Chronological age	0.538^{***}
	(0.080)
	t = 6.690
	p = 0.000
Sex	-2.810^{***}
	(0.997)
	t = -2.819
	p = 0.006
Visual age	0.180^{**}
	(0.074)
	t = 2.437
	p = 0.016
Constant	18.244^{***}
	(2.177)
	t = 8.379
	p = 0.000
Observations	154
R^2	0.695
Adjusted R^2	0.689
Residual Std. Error	5.867 (df = 150)
F Statistic	114.080^{***} (df = 3; 150) (p = 0.000)
Note:	*p<0.1; **p<0.05; ***p<0.01

Simulation of gene knockdowns with higher fold-change and multiple knockdowns

The log2 fold-change for the simulated gene knockdowns of all genes in the model was chosen to be in range of what would be realistically expected in expression differences in the context of “normal” physiological aging, therefore was set to a moderately strong effect size of -2. In order to test if observed effects on the age estimates, which were relatively small in magnitude, were also reproducible at higher magnitude, we have calculated the impacts for additional, stronger knockdowns (log2 fold-change = -10) as well, which showed concordantly stronger impact, but matching very well (R^2 = 0.98) with the previously reported directions for the individual gene knockdowns (Supplementary Figure 3 b). We also performed additional knockdowns of multiple pro-/anti-aging genes acting in a similar pathway or process. The experiments included the combined downregulation of the prominent aging target gene SIRT1 and the casein kinase coding gene CSNK2A2, which acts in the same pathways as a regulator of SIRT1 binding affinity (Supplementary Figure 3 c), the knockdown of PTEN and its direct regulator TP53 (Supplementary Figure 3 d), as well as the downregulation of the senescence markers CDKN2A

and SERPINE1 (Supplementary Figure 3 e). The combined knockdowns of all tested combinations showed accumulative effects when compared to single gene knockdowns.

Further model validation experiments

To illustrate the gain in reproducibility awarded by the utilization of an adapted loss function including a term optimizing the auxiliary output from the pathway neurons as well as the ensembling approach, we have added data showing the impact on reproducibility from a single network with and without the auxiliary loss operating on the pathway layer, as well the final ensemble model from separately retrained models (Supplementary Figure 4 a – c).

To ensure the functioning of the model as expected, we trained another model with a permuted filter matrix, in which gene-pathway affiliations were unchanged but the order of genes was permuted. The model's pathway ranking did not differ substantially from the original model (Supplementary Figure 4 d), indicating that interpretability was not impeded and the model was functioning as intended.

In order to ensure that the relatively even distribution of the pathway ranking in the model was not caused by the L2 regularization, retrained a model without L2 penalization to compare with the original model. As shown in Supplementary Figure 4 e, this model performed slightly worse on the test set in terms of precision than the regularized model. The comparison of the intermediate activations of the two models shows that the pathway ranking (Supplementary Figure 4 f) and distribution (Supplementary Figure 4 g) barely differ, indicating that the relatively even distribution observed in the pathway ranking of the original model is not a technical artifact caused by the regularization.

We also performed several experiments introducing control pathways into the model, artificial pathways consisting of varying numbers of randomly sampled genes (10, 25, 50, 100, 200). Analyzing the pathway activation ranking after retraining the model identified the control pathways as showing lower than average correlation to the modeled phenotype in all cases (Supplementary Figure 6 a). We then repeated the perturbation experiments using the photoaging, Hutchinson-Gilford-Progeria-Syndrome and caloric restriction signatures to ensure that interpretability of the model was not impeded by the addition of the additional pathways, which generated results comparable to the original model (Supplementary Figure 6 b – d), indicating that interpretability was not affected by the added control pathways. Overall model

accuracy was also not significantly altered (Supplementary Figure 6 e). One of the control pathways (control 5, the largest one consisting of 200 randomly sampled genes) ranked relatively high in the pathway ranking compared to the other control sets, presumably due to some correlative information present among the randomly sampled genes. In order to test this, we trained another model using a new set of control pathways consisting of randomly sampled genes with decidedly low associations to age (absolute Pearson correlation coefficient < 0.1). In concordance with the above mentioned hypothesis, these negative control pathways showed far lower correlation to the modeled phenotype (Supplementary Figure 7 a), indicating that the slightly better performance of the completely randomly sampled genes in the previous model was indeed driven by residual correlation present in the selected features. We also repeated the perturbation experiments using the HGPS/photoaging/caloric restriction signatures with this model and again found that the added pathways had no substantial impact on the interpretability of the model (Supplementary Figure 7 b – d) or model performance (Supplementary Figure 7 e).

In order to generate more thorough statistical estimates for the pathway ranking, we calculated 100 permutations on a network including a control pathway of 150 completely randomly chosen genes (matching the average size of the Hallmark pathways). The analysis of the resulting data using Wilcoxon rank sum tests comparing the Hallmark pathways to the introduced control showed that most of the pathways exhibited significantly stronger age association than the introduced baseline after multiple testing adjustment, with p53 signaling and several inflammatory pathways leading the list (Supplementary Table 2, same data as shown in Figure 2 d).

Supplementary Table 2: Statistics from comparing the correlation of pathway neuron activations of every pathway in the model to a control pathway of 150 randomly sampled genes using Wilcoxon rank sum tests, with data derived from 100 random permutations, consisting of retraining and testing the neural network ensemble:

	group1	group2	n1	n2	statistic	p	p.adj
1	control	p53 pathway	100	100	0	1.28e-34	6.4e-33
2	control	tnfa signaling via nfkb	100	100	0	1.28e-34	6.4e-33
3	control	uv response dn	100	100	7	1.58e-34	7.584e-33
4	control	apoptosis	100	100	10	1.73e-34	8.131e-33
5	control	interferon gamma response	100	100	18	2.2e-34	1.012e-32
6	control	inflammatory response	100	100	25	2.71e-34	1.2195e-32
7	control	il2 stat5 signaling	100	100	68	9.75e-34	4.29e-32
8	control	allograft rejection	100	100	101	2.58e-33	1.1094e-31
9	control	myogenesis	100	100	106	2.99e-33	1.2558e-31
10	control	estrogen response late	100	100	110	3.37e-33	1.3817e-31
11	control	epithelial mesenchymal transition	100	100	129	5.88e-33	2.352e-31
12	control	peroxisome	100	100	131	6.23e-33	2.4297e-31
13	control	cholesterol homeostasis	100	100	159	1.41e-32	5.358e-31
14	control	hypoxia	100	100	159	1.41e-32	5.358e-31
15	control	fatty acid metabolism	100	100	190	3.47e-32	1.2492e-30
16	control	uv response up	100	100	192	3.68e-32	1.288e-30
17	control	myc targets v1	100	100	194	3.89e-32	1.3226e-30
18	control	il6 jak stat3 signaling	100	100	199	4.5e-32	1.485e-30
19	control	mtorc1 signaling	100	100	201	4.77e-32	1.5264e-30
20	control	heme metabolism	100	100	266	3.07e-31	9.517e-30
21	control	mitotic spindle	100	100	332	1.98e-30	5.94e-29
22	control	e2f targets	100	100	439	3.87e-29	1.1223e-27
23	control	androgen response	100	100	494	1.74e-28	4.872e-27
24	control	interferon alpha response	100	100	573	1.45e-27	3.915e-26
25	control	apical junction	100	100	610	3.87e-27	1.0062e-25
26	control	oxidative phosphorylation	100	100	708	5.02e-26	1.255e-24
27	control	kras signaling up	100	100	848	1.77e-24	4.248e-23
28	control	complement	100	100	919	1.03e-23	2.369e-22
29	control	coagulation	100	100	967	3.33e-23	7.326e-22
30	control	angiogenesis	100	100	1162	3.41e-21	7.161e-20
31	control	adipogenesis	100	100	1631	9.32e-17	1.864e-15
32	control	g2m checkpoint	100	100	1700	3.75e-16	7.125e-15
33	control	tgf beta signaling	100	100	1823	4.2e-15	7.56e-14
34	control	estrogen response early	100	100	2237	7.4e-12	1.258e-10
35	control	protein secretion	100	100	2355	5.18e-11	8.288e-10
36	control	glycolysis	100	100	2389	8.94e-11	1.341e-09
37	control	dna repair	100	100	2604	2.41e-09	3.374e-08
38	control	apical surface	100	100	2645	4.38e-09	5.694e-08
39	control	kras signaling dn	100	100	2772	2.62e-08	3.144e-07
40	control	unfolded protein response	100	100	3207	5.94e-06	6.534e-05
41	control	reactive oxygen species pathway	100	100	3480	0.000103	0.00103
42	control	pi3k akt mtor signaling	100	100	4388	0.068	0.612
43	control	bile acid metabolism	100	100	4658	0.202	1
44	control	hedgehog signaling	100	100	5957	0.99	1
45	control	myc targets v2	100	100	8898	1	1
46	control	notch signaling	100	100	7761	1	1
47	control	pancreas beta cells	100	100	10000	1	1
48	control	spermatogenesis	100	100	7046	1	1
49	control	wnt beta catenin signaling	100	100	8670	1	1
50	control	xenobiotic metabolism	100	100	4565	0.144	1

Supplementary Figures:

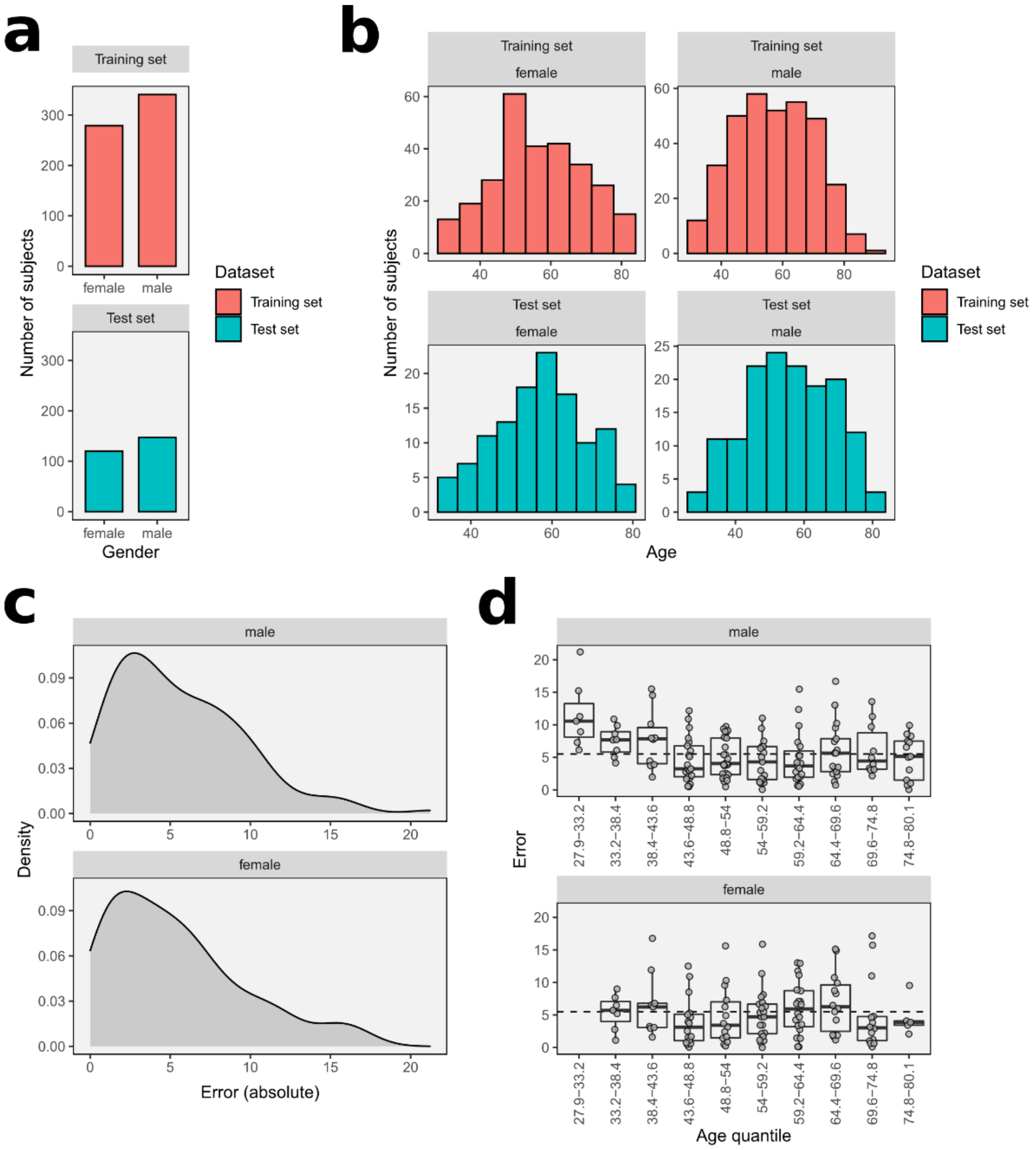

Supplementary Figure 1: **Gender and age distributions of training and test sets**: (a) Gender distribution in training and test set. (b) Histograms showing the age distributions in training and test set, split by gender. (c) Distributions of absolute prediction errors for the test set, split by gender. (d) Distribution of errors for the test set for different age quantiles and split by gender.

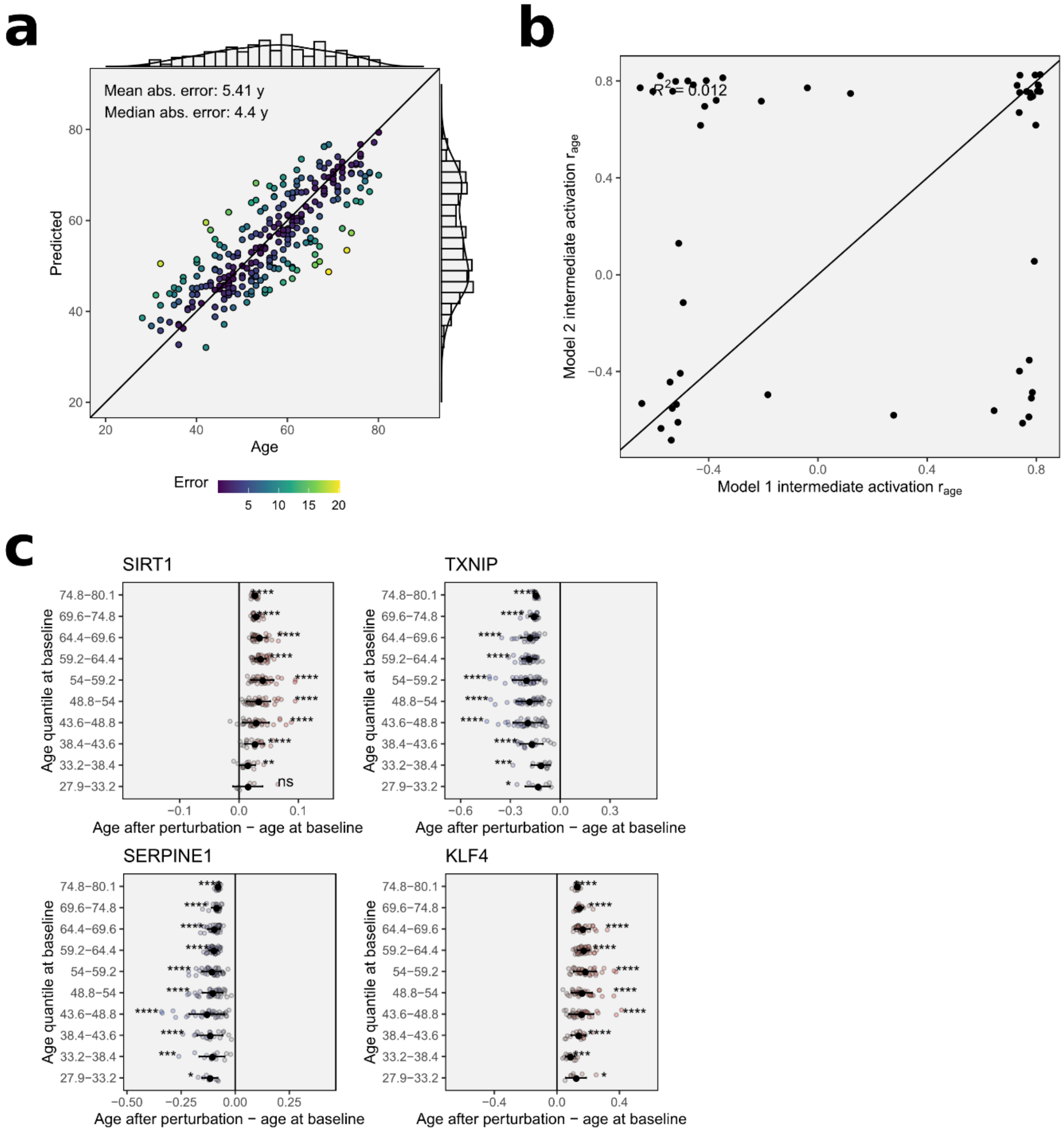

Supplementary Figure 2: **Fully connected "black box" neural network**: (a) Predicted against actual chronological age for the held out test set, with observations colored by absolute prediction error from an ensemble of 10 fully connected neural networks. (b) Comparison of the correlation of intermediate neuron activations to the modeled phenotype in the final hidden layer before the output layer for two neural networks from the final ensemble. (c) Predicted effects of the *in silico* knockdowns of SIRT1, TXNIP, SERPINE1 and KLF4 using the fully connected neural network ensemble. Effect on age is stratified by chronological age quantiles of the test subjects. Significance was determined using one-sample Wilcoxon rank sum tests, testing for the difference in medians from an effect size of 0, with p-values adjusted for multiple testing. Error bars show standard deviations.

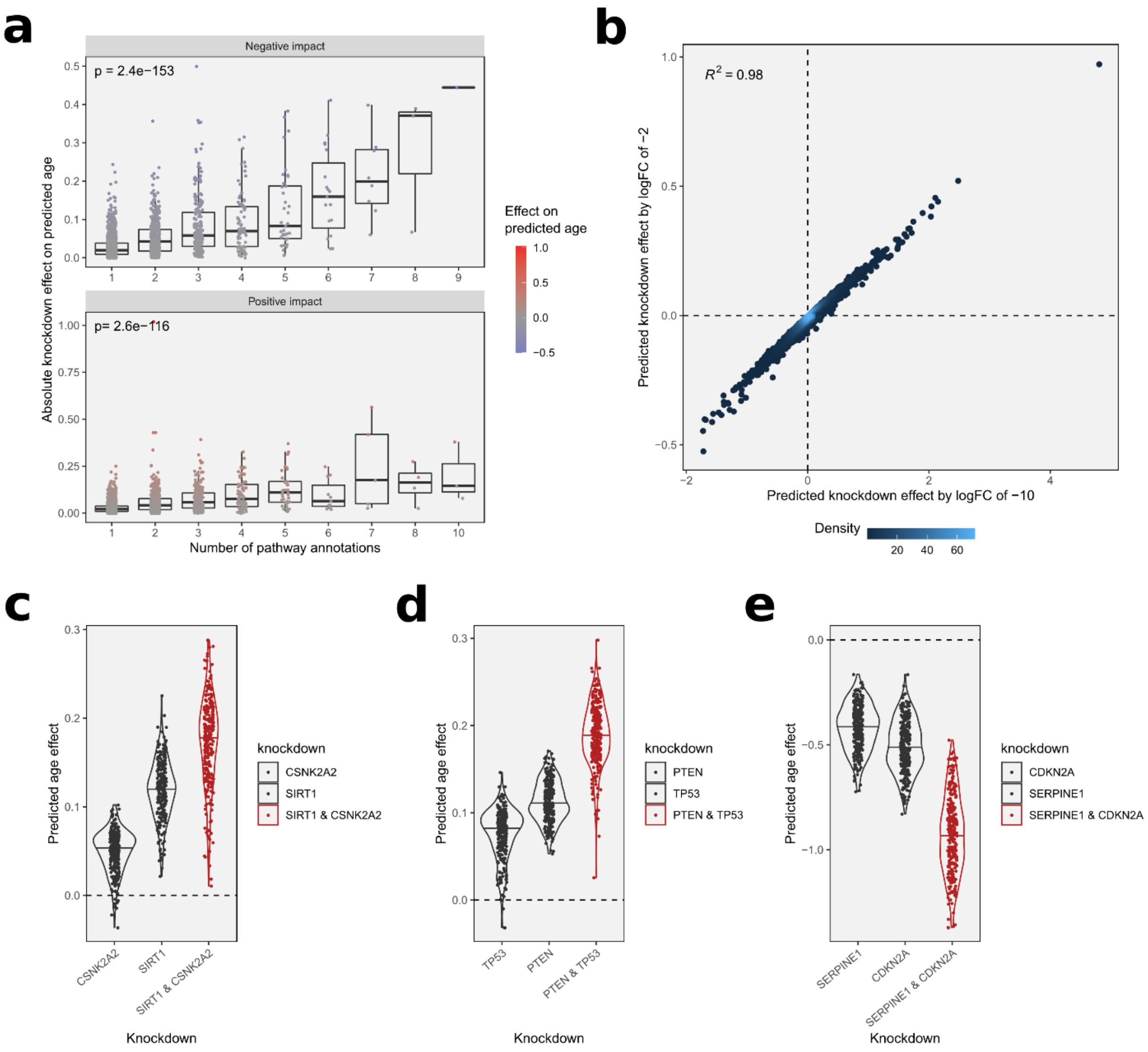

Supplementary Figure 3: **Further characterization of simulated gene knockdowns**: (a) Increased impact of genes at the crossroads of multiple pathways. The plot shows the predicted effect of gene knockdowns, stratified by the number of pathways each gene is annotated to. The plot is split by negative impact (upper panel) and positive impact on age prediction (lower panel). (b) Concordance between the directions of predicted impacts for gene knockdowns with log2 fold-changes of -2 and -10 for all genes in the model. (c) Comparison of the combined knockdown of SIRT1 and CSNK2A2 to single knockdowns of the two genes. (d) Comparison of the combined knockdown of PTEN and TP53 to single knockdowns of the two genes. (e) Comparison of the combined knockdown of SERPINE1 and CDKN2A to single knockdowns of the two genes.

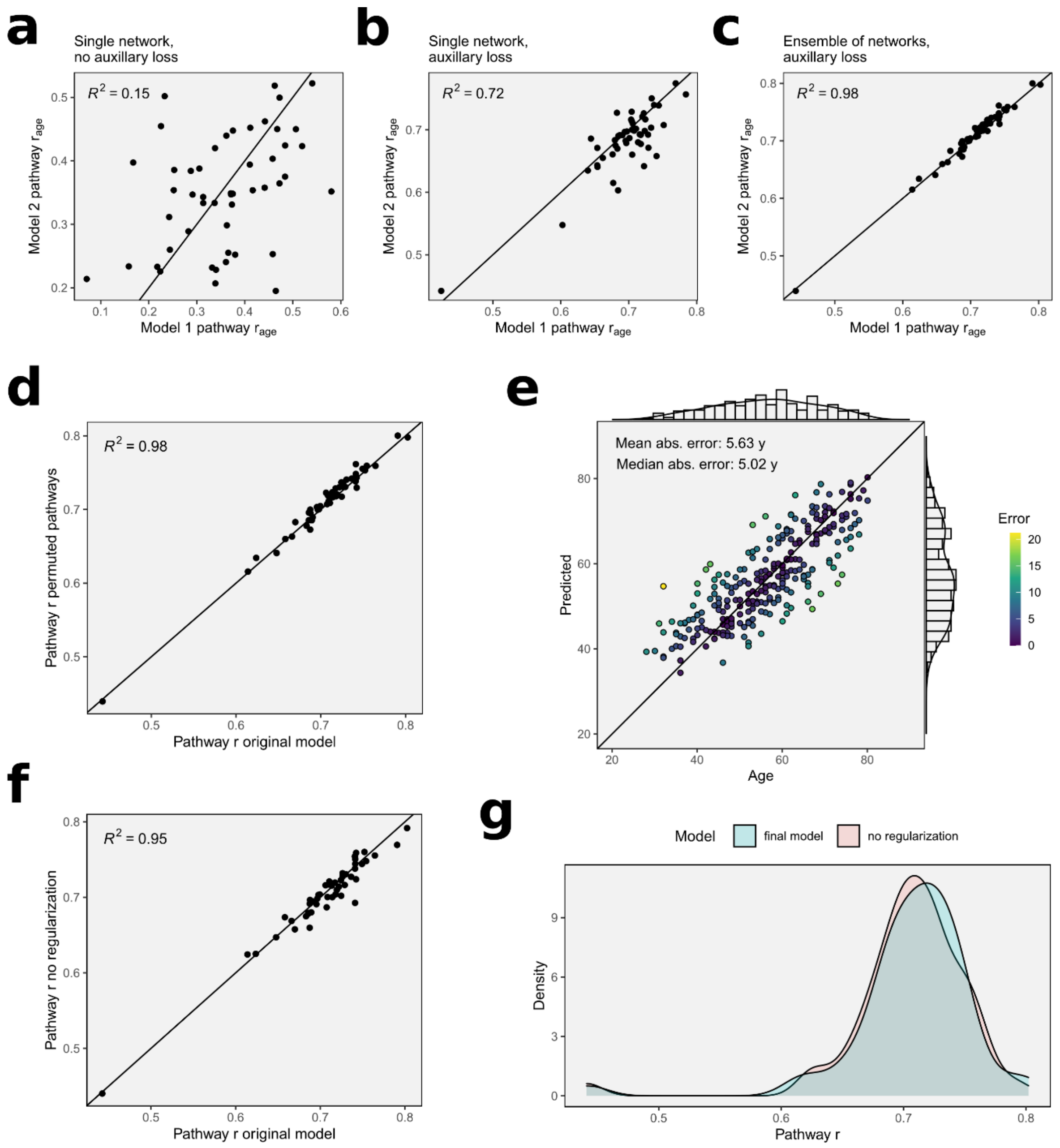

Supplementary Figure 4: **Validation experiments assessing the reproducibility of pathway neuron activations**: (a) Concordance between pathway rankings based on correlation of neuron activations and age for two single networks trained without auxiliary loss term. (b) Concordance between pathway rankings based on correlation of neuron activations and age for two single networks trained with the additional auxiliary loss term optimizing pathway neuron activations. (c) Concordance between pathway rankings based on correlation of neuron activations and age for two ensemble models consisting of 10 networks trained with the additional auxiliary loss term optimizing pathway neuron activations. (d) Concordance between pathway rankings of the original model and a newly trained model in which

the sequence of genes was permuted (gene-pathway affiliations kept unchanged). (e) Predicted against actual chronological age by a model retrained without L2 regularization, with observations colored by absolute prediction error. (f) Concordance between pathway rankings of the original model and the model without L2 regularization. (g) Distributions of pathway rankings based on correlation of neuron activations and age for the original model and the model without L2 regularizations.

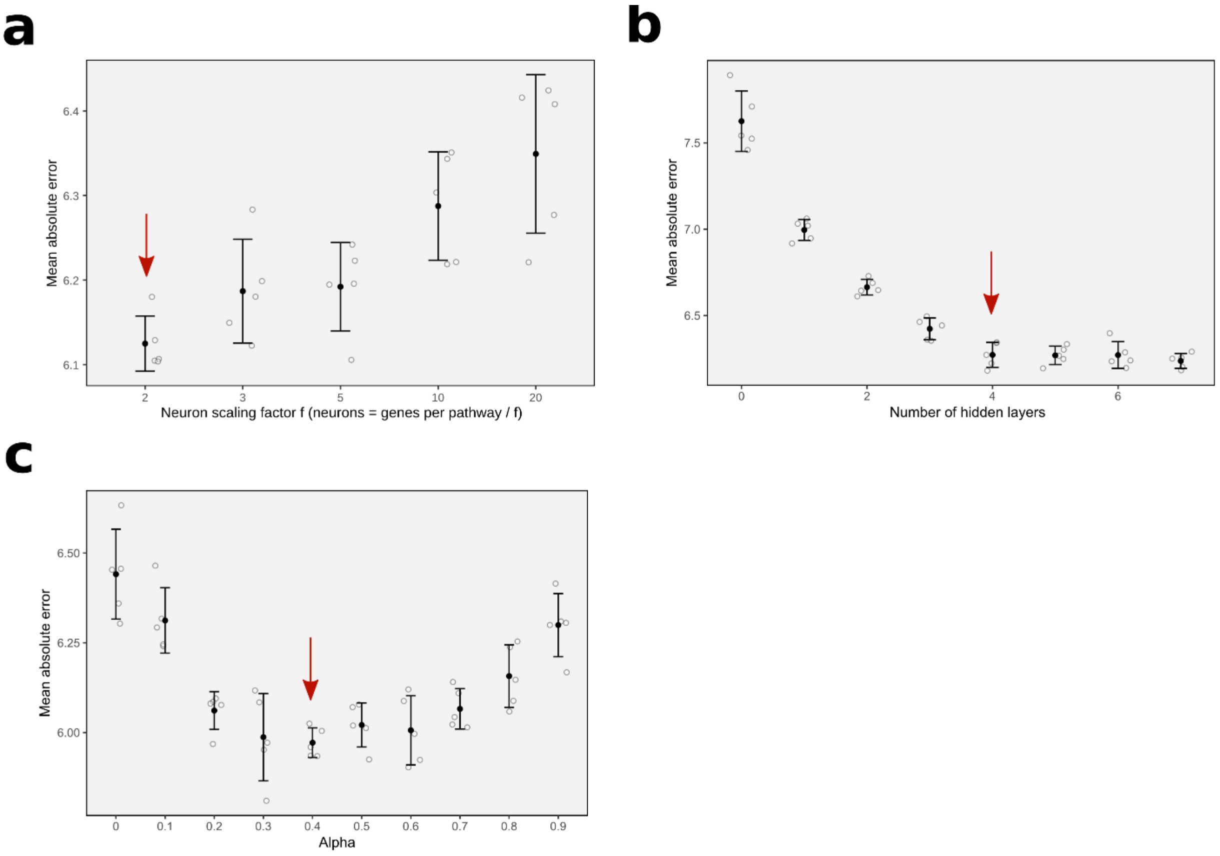

Supplementary Figure 5: **Model hyperparameters**: (a) Impact of the relative number of neurons per hidden layer on model accuracy. The number of neurons in each layer was adjusted to the number of genes in a given pathway to compensate for the increase in regulation complexity, the amount of which was determined by the neuron scaling factor *f*. The final model used a scaling factor of 2, i.e. every two additional genes awarded an additional neuron to the pathway layer instance. Error bars show standard deviations. (b) Impact of the number of hidden layers in the network on model accuracy. The final model used 4 hidden layers, as no substantial improvement in accuracy offset the increase in complexity thereafter. Error bars show standard deviations. (c) Impact of different configurations of hyperparameter alpha for balancing the two parts of the loss function – the main loss and the auxiliary loss from the

pathway layer outputs – during model training. The final model used an alpha of 0.4. Error bars show standard deviations.

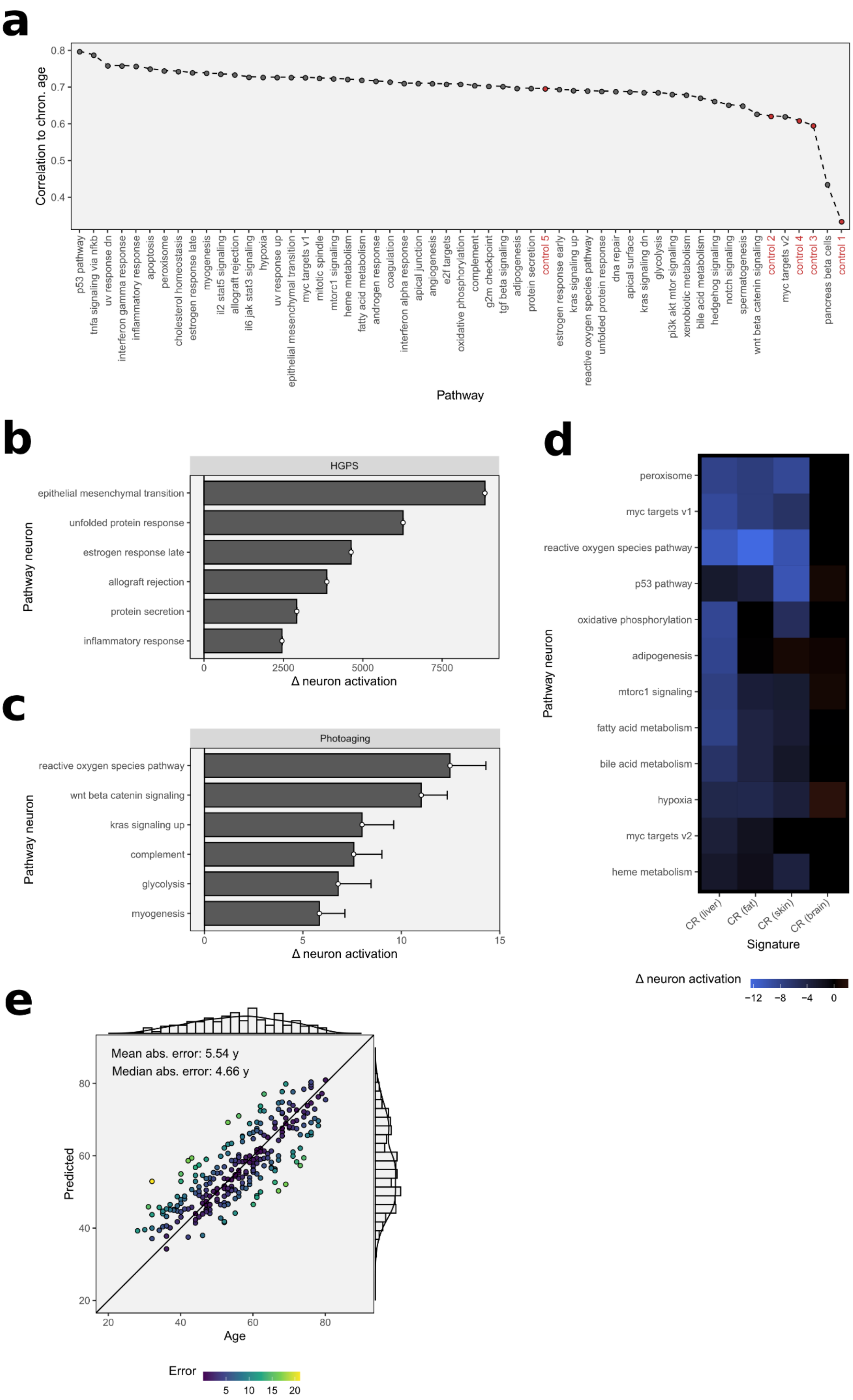
a
Correlation to chron. age
0.8
0.7
0.6
0.5
0.4
p53 pathway
tnfa signaling via nfkb
uv response dn
interferon gamma response
inflammatory response
apoptosis
peroxisome
cholesterol homeostasis
estrogen response late
myogenesis
il2 stat5 signaling
allograft rejection
il6 jak stat3 signaling
hypoxia
uv response up
epithelial mesenchymal transition
myc targets v1
mitotic spindle
mtorc1 signaling
heme metabolism
fatty acid metabolism
androgen response
coagulation
interferon alpha response
apical junction
angiogenesis
e2f targets
oxidative phosphorylation
complement
g2m checkpoint
tgf beta signaling
adipogenesis
protein secretion
control 5
estrogen response early
kras signaling up
reactive oxygen species pathway
unfolded protein response
dna repair
apical surface
kras signaling dn
glycolysis
pi3k akt mtor signaling
xenobiotic metabolism
bile acid metabolism
hedgehog signaling
notch signaling
spermatogenesis
wnt beta catenin signaling
control 2
myc targets v2
control 4
control 3
pancreas beta cells
control 1
Pathway
b
HGPS
Pathway neuron
epithelial mesenchymal transition
unfolded protein response
estrogen response late
allograft rejection
protein secretion
inflammatory response
0
2500
5000
7500
Δ neuron activation
c
Photoaging
Pathway neuron
reactive oxygen species pathway
wnt beta catenin signaling
kras signaling up
complement
glycolysis
myogenesis
0
5
10
15
Δ neuron activation
d
Pathway neuron
peroxisome
myc targets v1
reactive oxygen species pathway
p53 pathway
oxidative phosphorylation
adipogenesis
mtorc1 signaling
fatty acid metabolism
bile acid metabolism
hypoxia
myc targets v2
heme metabolism
CR (liver)
CR (fat)
CR (skin)
CR (brain)
Signature
Δ neuron activation
−12
−8
−4
0
e
Mean abs. error: 5.54 y
Median abs. error: 4.66 y
Predicted
80
60
40
20
20
40
60
80
Age
Error
5
10
15
20

Supplementary Figure 6: **Model validation using control pathways with randomly sampled genes**: (a) Pathway ranking based on Pearson correlation coefficients of pathway neuron activations and chronological age over the test set with control pathways consisting of 10, 25, 50, 100 and 200 randomly chosen, unrelated genes. (b) Effect of the transcriptional signature of Hutchinson-Gilford progeria syndrome on pathway neuron activation of a model containing the control pathways. Error bars show standard deviations. (c) Effect of the transcriptional signature of photoaging on pathway neuron activation of a model containing the control pathways. Error bars show standard deviations. (d) Heatmap showing the effects of tissue-specific caloric restriction signatures on pathway neuron activation of a model containing the control pathways. (e) Predicted against actual chronological age for the held out test set by a model containing the control pathways, with observations colored by absolute prediction error.

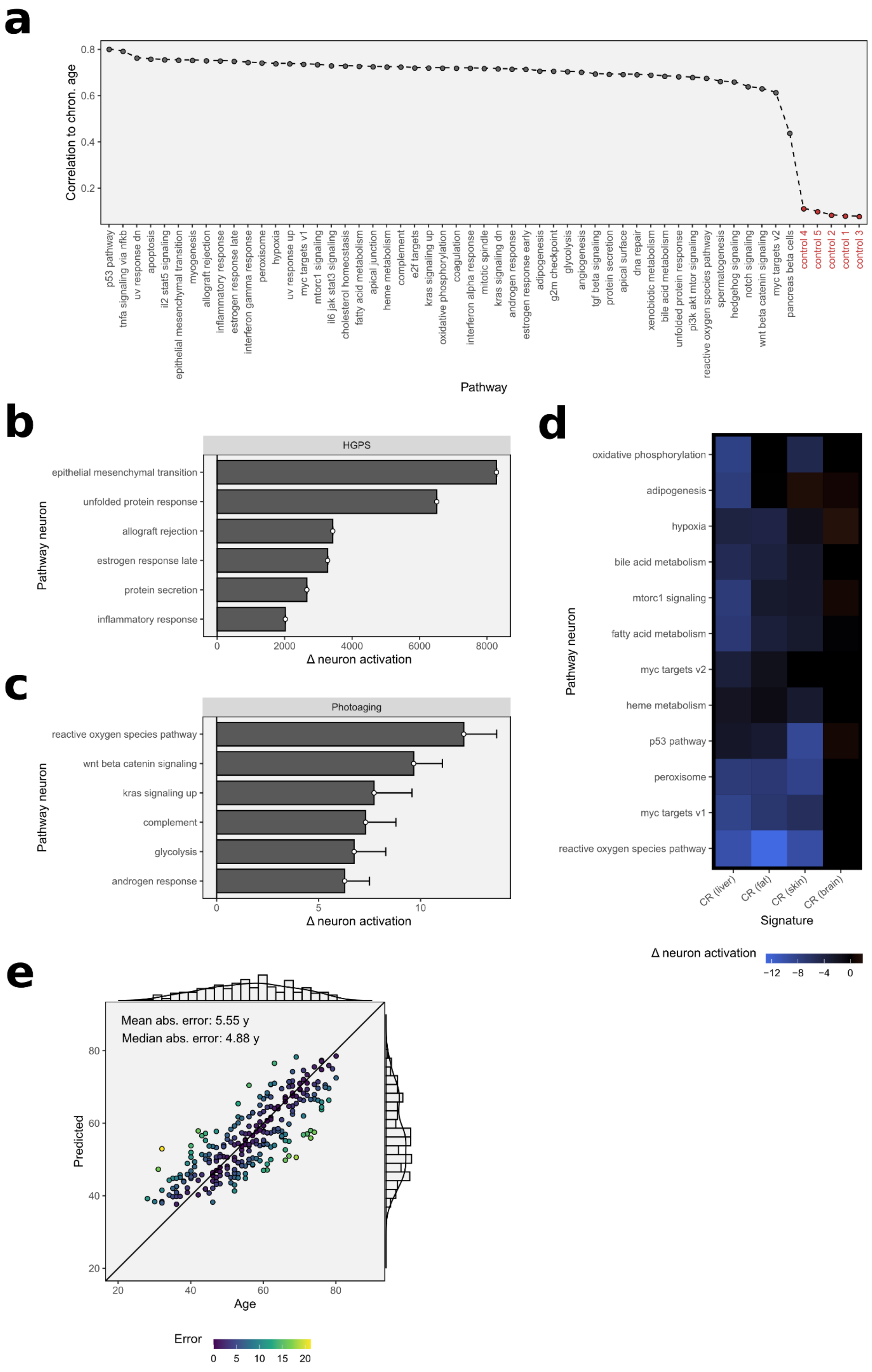
a
Correlation to chron. age
0.8
0.6
0.4
0.2
p53 pathway
tnfa signaling via nfkb
uv response dn
apoptosis
il2 stat5 signaling
epithelial mesenchymal transition
myogenesis
allograft rejection
inflammatory response
estrogen response late
interferon gamma response
peroxisome
hypoxia
uv response up
myc targets v1
mtorc1 signaling
il6 jak stat3 signaling
cholesterol homeostasis
fatty acid metabolism
apical junction
heme metabolism
complement
e2f targets
kras signaling up
oxidative phosphorylation
coagulation
interferon alpha response
mitotic spindle
kras signaling dn
androgen response
estrogen response early
adipogenesis
g2m checkpoint
glycolysis
angiogenesis
tgf beta signaling
protein secretion
apical surface
dna repair
xenobiotic metabolism
bile acid metabolism
unfolded protein response
pi3k akt mtor signaling
reactive oxygen species pathway
spermatogenesis
hedgehog signaling
notch signaling
wnt beta catenin signaling
myc targets v2
pancreas beta cells
control 4
control 5
control 2
control 1
control 3
Pathway
b
HGPS
Pathway neuron
epithelial mesenchymal transition
unfolded protein response
allograft rejection
estrogen response late
protein secretion
inflammatory response
0
2000
4000
6000
8000
Δ neuron activation
c
Photoaging
Pathway neuron
reactive oxygen species pathway
wnt beta catenin signaling
kras signaling up
complement
glycolysis
androgen response
0
5
10
Δ neuron activation
d
Pathway neuron
oxidative phosphorylation
adipogenesis
hypoxia
bile acid metabolism
mtorc1 signaling
fatty acid metabolism
myc targets v2
heme metabolism
p53 pathway
peroxisome
myc targets v1
reactive oxygen species pathway
CR (liver)
CR (fat)
CR (skin)
CR (brain)
Signature
Δ neuron activation
−12
−8
−4
0
e
Mean abs. error: 5.55 y
Median abs. error: 4.88 y
Predicted
80
60
40
20
20
40
60
80
Age
Error
0
5
10
15
20

Supplementary Figure 7: **Model validation using control pathways with randomly sampled genes exhibiting low age correlations**: (a) Pathway ranking based on Pearson correlation coefficients of pathway neuron activations and chronological age over the test set with control pathways consisting of 10, 25, 50, 100 and 200 randomly chosen, unrelated genes with low age correlation (absolute Pearson correlation coefficients below 0.1). (b) Effect of the transcriptional signature of Hutchinson-Gilford progeria syndrome on pathway neuron activation of a model containing the control pathways. Error bars show standard deviations. (c) Effect of the transcriptional signature of photoaging on pathway neuron activation of a model containing the control pathways. Error bars show standard deviations. (d) Heatmap showing the effects of tissue-specific caloric restriction signatures on pathway neuron activation of a model containing the control pathways. (e) Predicted against actual chronological age for the held out test set by a model containing the control pathways, with observations colored by absolute prediction error.
