# Supplementary material for: Modeling transcriptomic age using knowledge-primed artificial neural networks
Source: NPJ Aging Mech Dis. 2021 Jun 1;7:15. doi: 10.1038/s41514-021-00068-5 (PMC8169742; doi:10.1038/s41514-021-00068-5)
Supplement: Supplementary file 2 — Reporting Summary [file 41514_2021_68_MOESM2_ESM.pdf]

nature research

Corresponding author(s): Nicholas Holzscheck
Lars Kaderali

Last updated by author(s): Apr 16, 2021

Reporting Summary

Nature Research wishes to improve the reproducibility of the work that we publish. This form provides structure for consistency and transparency in reporting. For further information on Nature Research policies, see our Editorial Policies and the Editorial Policy Checklist.

Statistics

For all statistical analyses, confirm that the following items are present in the figure legend, table legend, main text, or Methods section.

n/a	Confirmed	
☐	☒	The exact sample size (*n*) for each experimental group/condition, given as a discrete number and unit of measurement
☒	☐	A statement on whether measurements were taken from distinct samples or whether the same sample was measured repeatedly
☐	☒	The statistical test(s) used AND whether they are one- or two-sided<br>*Only common tests should be described solely by name; describe more complex techniques in the Methods section.*
☐	☒	A description of all covariates tested
☐	☒	A description of any assumptions or corrections, such as tests of normality and adjustment for multiple comparisons
☐	☒	A full description of the statistical parameters including central tendency (e.g. means) or other basic estimates (e.g. regression coefficient) AND variation (e.g. standard deviation) or associated estimates of uncertainty (e.g. confidence intervals)
☐	☒	For null hypothesis testing, the test statistic (e.g. *F*, *t*, *r*) with confidence intervals, effect sizes, degrees of freedom and *P* value noted<br>*Give P values as exact values whenever suitable.*
☒	☐	For Bayesian analysis, information on the choice of priors and Markov chain Monte Carlo settings
☒	☐	For hierarchical and complex designs, identification of the appropriate level for tests and full reporting of outcomes
☐	☒	Estimates of effect sizes (e.g. Cohen's *d*, Pearson's *r*), indicating how they were calculated

Our web collection on statistics for biologists contains articles on many of the points above.

Software and code

Policy information about availability of computer code

Data collection	n/a
Data analysis	Fastqc v0.11.7, Trimmomatic v0.36, Salmon v0.8.1, and R v3.6.1 including the packages tensorflow, keras, data.table, dplyr, rtsne, ggplot2 and ggpubr

For manuscripts utilizing custom algorithms or software that are central to the research but not yet described in published literature, software must be made available to editors and reviewers. We strongly encourage code deposition in a community repository (e.g. GitHub). See the Nature Research guidelines for submitting code & software for further information.

Data

Policy information about availability of data

All manuscripts must include a data availability statement. This statement should provide the following information, where applicable:

- Accession codes, unique identifiers, or web links for publicly available datasets
- A list of figures that have associated raw data
- A description of any restrictions on data availability

All data generated in this study is available from the SHIP consortium upon official request (http://www2.medizin.uni-greifswald.de/cm/fv/ship/daten-beantragen/).

Field-specific reporting

Please select the one below that is the best fit for your research. If you are not sure, read the appropriate sections before making your selection.

☒ Life sciences ☐ Behavioural & social sciences ☐ Ecological, evolutionary & environmental sciences

For a reference copy of the document with all sections, see nature.com/documents/nr-reporting-summary-flat.pdf

Life sciences study design

All studies must disclose on these points even when the disclosure is negative.

Sample size	No dedicated sample size calculations were performed, however sample size greatly exceeds that of previously efforts to generate transcriptomic age clocks.
Data exclusions	No final data points were excluded from the analysis.
Replication	Training / test splits of the dataset confirmed the reproducibility of the model.
Randomization	All sampling was performed randomly during this study.
Blinding	Blinding was performed where appropriate (e.g. visual age estimation).

Reporting for specific materials, systems and methods

We require information from authors about some types of materials, experimental systems and methods used in many studies. Here, indicate whether each material, system or method listed is relevant to your study. If you are not sure if a list item applies to your research, read the appropriate section before selecting a response.

Materials & experimental systems

n/a	Involved in the study	
☒	☐	Antibodies
☒	☐	Eukaryotic cell lines
☒	☐	Palaeontology and archaeology
☒	☐	Animals and other organisms
☐	☒	Human research participants
☒	☐	Clinical data
☒	☐	Dual use research of concern

Methods

n/a	Involved in the study	
☒	☐	ChIP-seq
☒	☐	Flow cytometry
☒	☐	MRI-based neuroimaging

Human research participants

Policy information about studies involving human research participants

Population characteristics	As the SHIP study is an unbiased population-based study, all samples collected represented the natural distribution of age, gender etc. of the habitants of the Federal State of Mecklenburg/West Pomerania in Northeastern Germany.
Recruitment	Population-based recruitment of study participants is detailed elsewhere (DOI: 10.1093/ije/dyp394).
Ethics oversight	The study was approved by the ethics committee of the University Medicine Greifswald (ethics approval number BB 39/08).

Note that full information on the approval of the study protocol must also be provided in the manuscript.
